# High Sensitive Methods for Health Monitoring of Compressor Blades and Fatigue Detection

**DOI:** 10.1155/2013/218460

**Published:** 2013-09-29

**Authors:** Mirosław Witoś

**Affiliations:** Air Force Institute of Technology (AFIT), Ks. Bolesława 6, 01-494 Warszawa, Poland

## Abstract

The diagnostic and research aspects of compressor blade fatigue detection have been elaborated in the paper. The real maintenance and overhaul problems and characteristic of different modes of metal blade fatigue (LCF, HCF, and VHCF) have been presented. The polycrystalline defects and impurities influencing the fatigue, along with their related surface finish techniques, are taken into account. The three experimental methods of structural health assessment are considered. The metal magnetic memory (MMM), experimental modal analysis (EMA) and tip timing (TTM) methods provide information on the damage of diagnosed objects, for example, compressor blades. Early damage symptoms, that is, magnetic and modal properties of material strengthening and weakening phases (change of local dislocation density and grain diameter, increase of structural and magnetic anisotropy), have been described. It has been proven that the shape of resonance characteristic gives abilities to determine if fatigue or a blade crack is concerned. The capabilities of the methods for steel and titanium alloy blades have been illustrated in examples from active and passive experiments. In the conclusion, the MMM, EMA, and TTM have been verified, and the potential for reliable diagnosis of the compressor blades using this method has been confirmed.

## 1. Introduction

**Figure 1:**
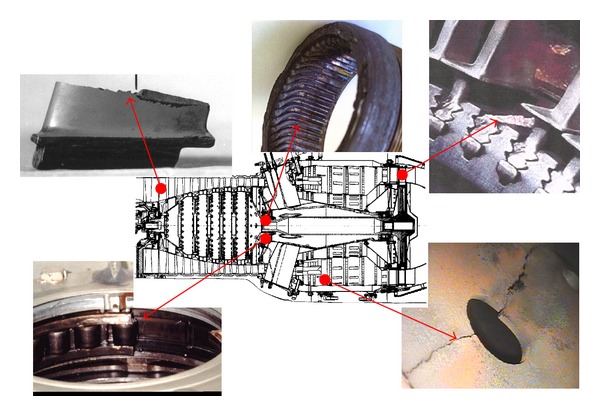
Problems of structural fatigue in turbine engines.

Many different fatigue failures,

- low cycle fatigue (LCF)
- high cycle fatigue (HCF),
- very high cycle fatigue (VHCF),
- thermomechanical fatigue (TMF),

could occur throughout the turbine engine's life (Figure 1). Most of them are damage to compressor blades described in the paper. 

**Figure 2:**
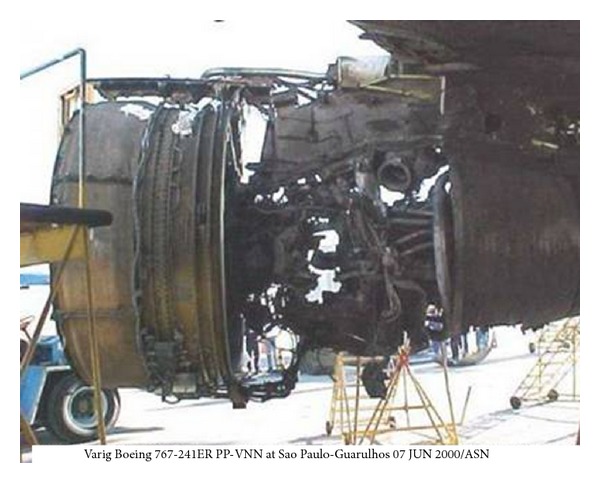
CF6-80 engine: fatigue destruction of III–IX stages of HP compressor [24].

Fatigue cracks propagating in rotor blades, the incorrect control of the engine's fuel system, and the lack of knowledge on the loads affecting the bearing system generally could cause formidable hazard to flight safety (Figure 2), as well as to engine life and reliability. Therefore, the AFIT keeps looking for new methods to recognize engineering, manufacturing, overhaul and service errors as well as stochastic overloads during the engine's running. Recognition of operational and maintenance problems is the first step to actively diagnose and control fatigue progress (engine's structural durability and reliability) as well as for prolongation engine time between overhaul and TBO [1–3]. 

The paper presents three diagnostic methods.

- *A metal magnetic memory* [4–7], which has been used as a sensitive passive observer of residual and applied stresses and material damages (of ferromagnetic and some austenite parts) [8–15]. Nowadays in the world MMM method has been used only for NDT of industrial objects, for example, welding joints, gas and steam turbine parts, rope, underground pipes, supporting structures in order to increase their TBO. The method has been tested in Poland for aviation (as NDE and SHM applications) since 2008 [3, 15, 16].
- *An experimental modal analysis* [17–21], which has been used in Poland as a sensitive NDE method during overhaul blade tests since 2008 and the method of the high-frequency identification of blade modal properties [3, 22, 23].
-  
*A tip timing method* [25–33], which is one of the most interesting methods of complex diagnosing of jet engines and a powerful tool in order to investigate dynamic phenomena. The method has been used in the Polish Air Force since 1993 with the SNDŁ-1b/SPŁ-2b diagnosing system developed for the SO-3 jet engines. Since 1997, this method has been also used in Poland in the postrepair/postoverhaul acceptance tests. Now, the method is developed as digital board computer for structural health monitoring (SHM) in different aero-engines. Roots of the method date back to years 20 of the 20th century, when it was drawn up in the analogue version by Sir Campbell to needs of examining vibration of steam turbine blades [34]. At present the method is dynamically elaborated and used in the world, mainly in aviation, in the digital version. Standardizing works (ISA 107.1 subcommittee) are being conducted by partner of European Virtual Institute for Gas Turbine Instrumentation (EVI GTI) [35] and the Propulsion Instrumentation Working Group (PIWG) [36]. Organizations joining the largest producers of aero-engines, research and education units and companies of the metrological support. Details of the TTM application are being protected by numerous inventions, for example, [37–54].

**Figure 3:**
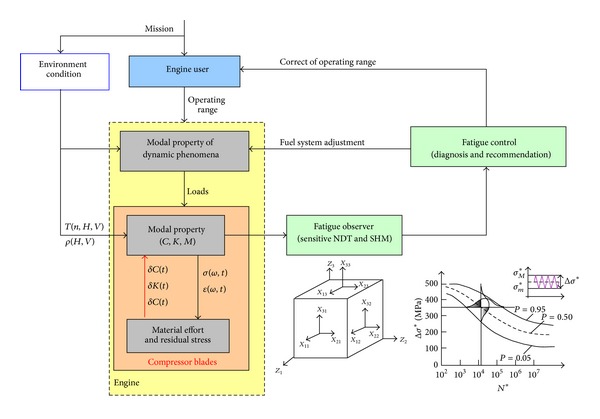
Idea of actively control fatigue by engine user—do not wait for blade crack [3].

The above described methods are sources of complex information about blade quality (of design, production, and overhaul) and real dynamics of phenomena correlated by modal properties, which have an effect on blades damage and fatigue differentiation. This information is used for holistic analysis of fatigue problems in aeroengines with influence of human and operating factors and actively control fatigue of compressor blade (Figure 3) as well as for verification of a FEM model.

## 2. Motivation

**Figure 4:**
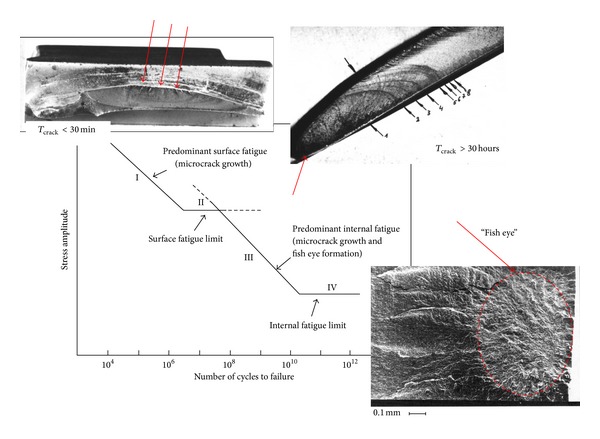
Fatigue problems (LCF, HCF, and VHCF) of compressor blades [3, 56].

In the years 1975–91 as many as 25 first-stage compressor blades of ten SO-3 jet engines suffered fatigue-attributable break-offs, which caused two accidents. The metallographic examination of damaged blades made out of the 18H2N4WA alloy structural steel has proved that the crack initiation zone was located either on the leading edges (55%) or on the blade-back surfaces (45%), in the areas of nodal lines of the first mode vibration. Crack propagation occurred at low-level stresses (HCF problem) or high-level stresses (LCF problem) (Figure 4). Fatigue fracture covering as much as 95% of the blade's cross section was found in one of the blades. Furthermore, it has also been found that erosion and corrosion, both occurring on the blade's face surface, as well as fine mechanical damages on the leading edge are stress concentrators [55]. Fatigue problem was also observed in titanium blades (Ti5.8Al-3.7Mo) in the TW3-117 engines in the years 2005–2007 [3].

The gigacycle fatigue of compressor blade (VHCF problem) with “fish eye” symptoms under the blade surface has been observed at foreign users, for example, in Russia [56, 57] (Figure 4). Compressor blades run a risk of VHCF problem because they count more than 3 · 10^9^ cycles for 1st flexible mode and more than 1 · 10^10^ cycles for 1st torsional mode during TBO. High risk of VHCF problem, with crack nucleation under the blade surface and stresses level below surface fatigue limits, concerns of high resistant material and blades made with surface finish techniques [58–60].

Uncontrolled blade over fatigue

- is a threat for service safety;
- limits aeroengine life time;
- increases maintenance costs.

**Figure 5:**
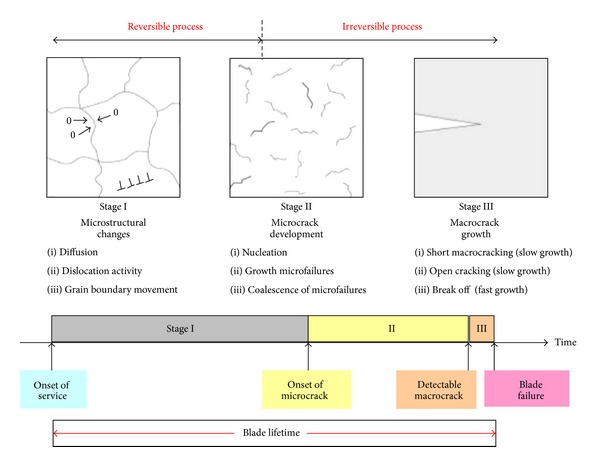
Continuum damage mechanics for compressor blade material. The first stage and the beginning of the second stage of damage are copying reversible changes [3].

It is also a great challenge for diagnostics engineers. Specialists, who are familiar with abilities of the NDE and SHM methods and continuum damage mechanics of material (Figure 5), are searching for answers to questions of the aero-engine user and the technologist of the renovation plant.

- *Level I*—before the first compressor blade crack or break-off
   
*Which types of aero-engine are unrecognized compressor fatigue problem on?*
             
*⇓*
   
*Is it possible to assess the fatigue risk of blades through the casing of the compressor?*
- *Level II*—technology and the quality of overhaul
   
*What methods of the compressor blades verification should apply in the overhaul so that the blade does not break in the guarantee period?*
             
*⇓*
   
*How to recognize overload and weakened compressor blades (2nd stage of damage)?*
- *Level III*—preventive activity in the overhaul and maintenance
   
*If the crack of the blade was detected, what is causing for fatigue problem?*
             
*⇓*
   
*What mistakes of the engine use and repair are affecting for precipitated fatigue of compressor blades?*

Classical NDE methods (X-ray, eddy current, ultrasound, magnetic particle and fluorescent) are very low effective ones for diagnosing blade crack before 3rd stage of damage because of the following.

**Figure 6:**
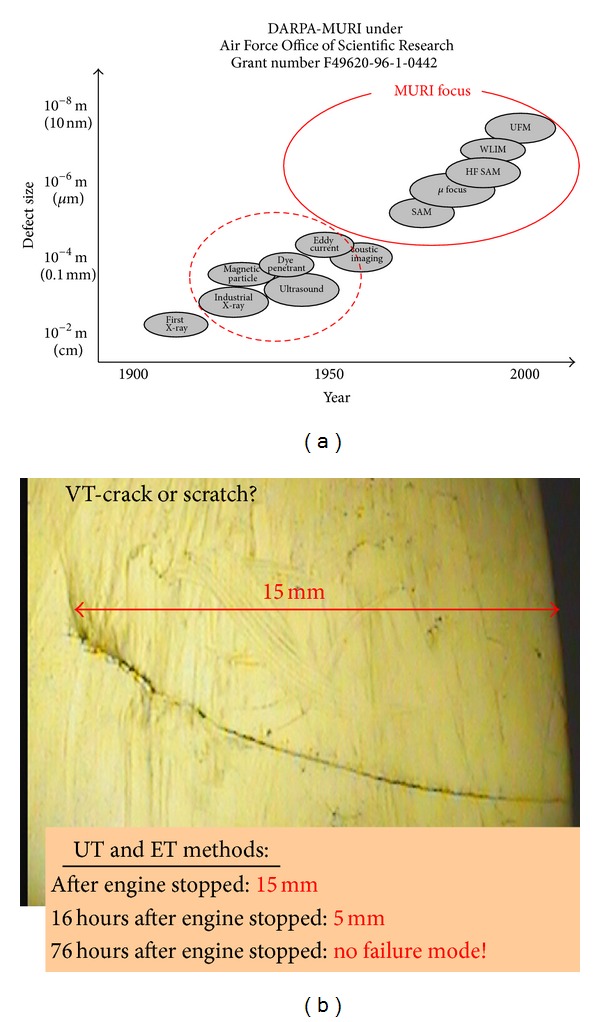
Showing: (a) Contemporary research capabilities of material degradation in service (the classic NDT methods) and the laboratory (MURI focus) [55]; (b) Influence residual stresses on closing the crack gap and the change of reading by ultrasound and eddy current methods after switching the engine off.

- They do not answer the questions mentioned above. Finding the crack or other discontinuity of the structure, rather than its causes, is their main task.
- Crack gap closing during engine standstill: Theoretical possibilities of the NDE method (detection of defects about min. size 0.3 mm) turn out to be false during examinations of the blades carried out after the longer stop of the engine, (Figure 6).
- Lack of reliable information about real operating conditions.
- Lack of knowledge about early cracking symptoms and mechanisms.
- Limited access to tested blades (because of inlet stator vane).

Other disadvantage of the NDE methods in use (during overhaul and service) is no possibility of *fatigue prognosis*.

**Figure 7:**
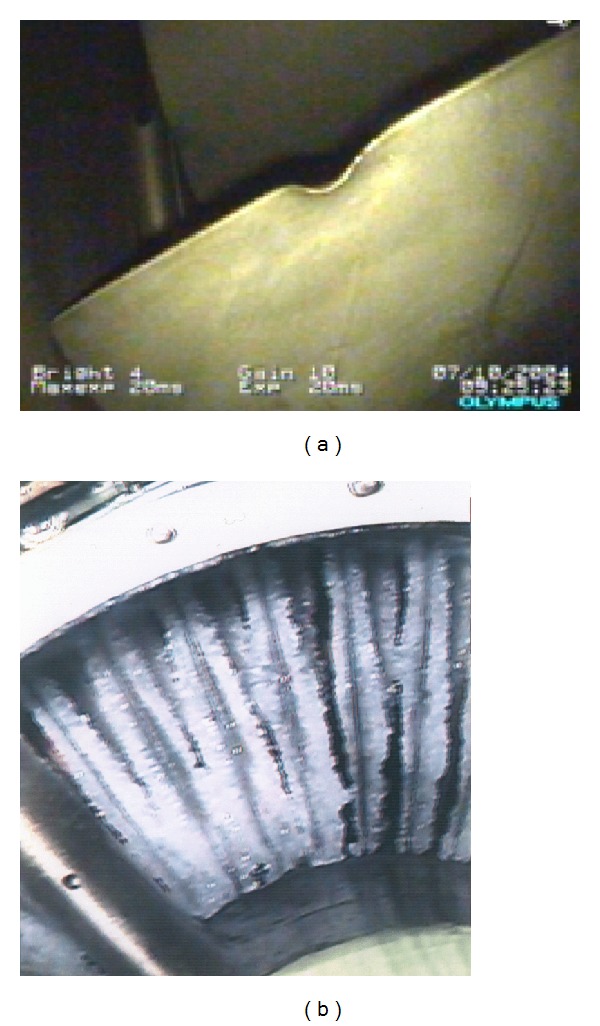
Showing LCF concentrations: (a) blade with FOD; (b) icing of compressor inlet.

This disadvantage is very important for blades having fatigue problems, for example, the 1st compressor blades of the SO-3 engine. These blades have design errors—too low the first-mode mistuning from the 2nd rotational harmonic excitation. Therefore, too high stress and fast fatigue crack initiation can occur during operation. These conditions take place during the take-off phase when there is a foreign object (e.g., bird) lying in the inlet or the inlet icing occurs (Figure 7). Under such conditions time between crack initiation and blade damage can be shorter than time of a single flight (LCF problem). Disturbance of the pressure and velocity in the intake are being moved by the entire length of the compressor. Hazardous blade vibrations occur only at synchronous resonance if the level of excitations is greater than acceptable. A diagram of Campbell is outlining zones of the synchronization, but it is well known only to a designer of the compressor. Flutter, surge (source of asynchronous resonances), and foreign object damage (FOD, local stress concentrator) are also source of LCF problems. Surge and FOD are easily detected by the aeroengine users.

**Figure 8:**
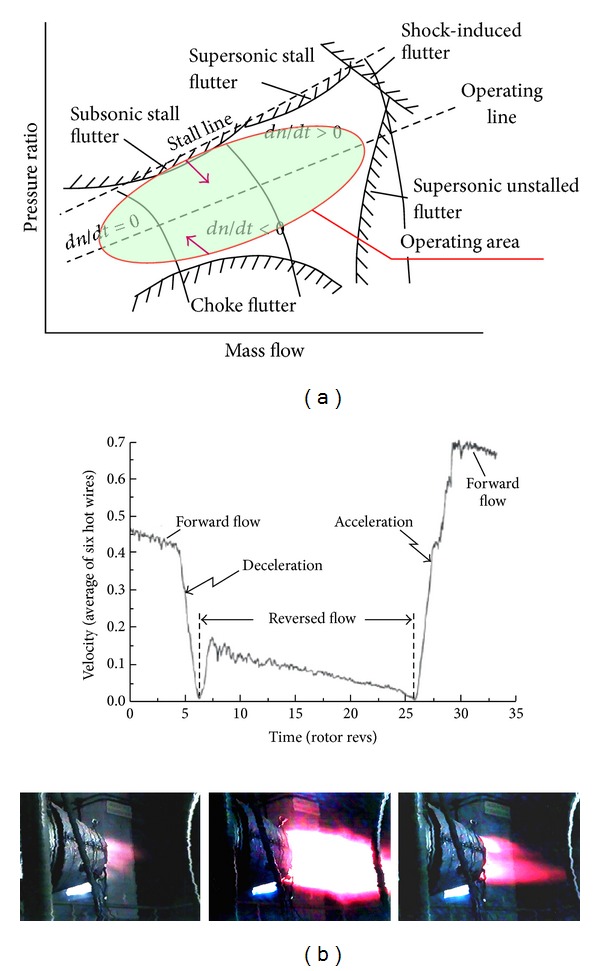
Showing (a) map of the compressor with information about dangerous threats [2, 3]; (b) deep surge cycle [3, 61]: the deceleration and acceleration of the flow in the compressor duct are a broadband impulse extortion for compressor blades and a bearing system (LCF concentrations) as well as with thermomechanical fatigue (TMF) for elements of a combustion chamber and the turbine.

Some errors of setting or hidden defects of the fuel control system affect the long-term working of compressor near the unstable limits (Figure 8) (stall, flutter, or surge during acceleration and deceleration, disturbance of the temperature field in compressor, combustion chamber and turbine), as well as high level of rotor unbalancing and alignment are the source of generating hidden fatigue problems of compressor blades (HCF and VHCF problems). Synchronous and asynchronous resonances are appearing in these working conditions of the compressor. The lack of distinct manifestations of the engine during disorders mentioned above causes that HCF and VHCF problems of blades, including tearing them off, are surprising for the user. Initiating the opened crack (3rd stage of damage) and its propagation are taking place at the low vibration amplitude of blades. Decreasing risk level of blades HCF and LCF is possible by correct overhaul and operation errors, that is, shape of operating area changes [2, 3, 61–63].

Endurance problems of compressor blades can result also from new acoustic properties of a combustion chamber and the combustion process, incurred after the change of the fuel type, for example, using Jet-A1 or F-34 (NATO) in place of Jet-B or during high disturbance of temperature field in front of the turbine generated by carbon deposit of injectors [3]. 

Both old and new properties of disadvantageous extortions are unknown for the aero-engines user. Endurance problem of the compressor blades is noticed only after the first coincidence of cracking or breaking off the blade in service.

**Figure 9:**
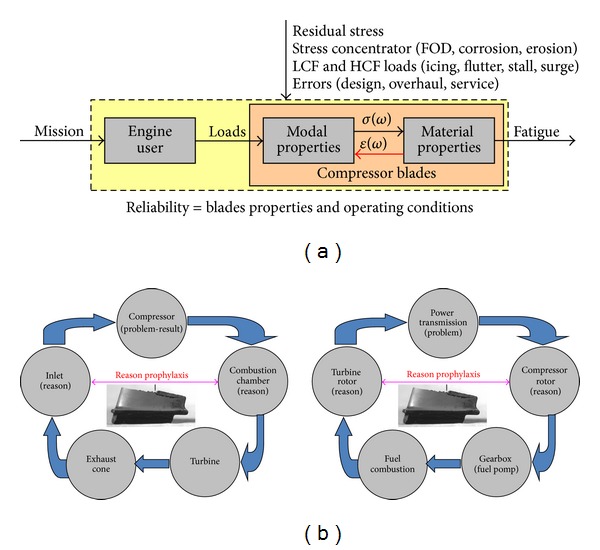
Analysis of cause and effect [3]: (a) classic approach with influence of human and operational factors on fatigue problems of the blades; (b) holistic approach—a compressor blade crack is a “*result*” of the wrong level of energy flow inside the engine. The “*causes*” must be sought at the inlet or the combustion chamber thermodynamic parameters (flow dynamics path) or mechanical power transmission quality and flight loads (kinematic loads path).

To sum up, fatigue problems of compressor blade are an effect rather than a cause. For the analysis of the problems, two approaches are used: classical and holistic. In *the classic approach*, the material fatigue results mainly from operating times (load cycles) and intensity of adverse phenomena (e.g., the human aspects and the specificity of aviation missions), Figure 9(a). In *the holistic approach* the material fatigue of compressor blade results from the level and the duration of disturbing the flow of the energy which aspects of the quality are affecting of the production, the repair and the use of the engine, Figure 9(b). The effective prevention requires applying the observer (the NDE and/or SHM method, methods of the signal analysis), which will be detecting not only the crack of blade, but also the cause of the hastened material fatigue (Table 1).

## 3. Concepts of Evaluating Blades

Effective and credible monitoring of the structure is possible, when technical problems are recognized, expected diagnostic symptoms are detected, and measuring and analytical methods (algorithms) could be applied.

### 3.1. Monitoring of the Vibration Magnitude

An intuitive diagnostic symptom of LCF problems is *blades magnitude.* Detecting the dangerous level of blade vibration during the work of an aeroengine and the change of flight conditions or the rotational speed of the engine are a base of the straightest preventive activity. 

A report is describing the diagnostic rule

(1) $$\begin{array}{c} \text{if}\;A_{\text{blades}}<A_{\max},\text{then OK else LCF threat.} \end{array}$$

This approach has advantages and disadvantages.

*Advantages*

- Practicable methods in the service and flight (for a board monitoring system);
- Possible active control of blades LCF.

*Disadvantages*

- The level of the dangerous vibration amplitude is different for different mod (higher mod *⇒* smaller acceptance amplitude);
- The indicator of the dangerous vibration amplitude is not detecting the HCF and VHCF threat of blades;
- During the work of an engine, the vibration amplitude of the cracked blade little differs from healthy blades (influence of the centrifugal force).

### 3.2. Monitoring of the Mod Frequency

An intuitive diagnostic symptom of a blade crack is “a in change its modes frequency” (influence of active area change). The cracking propagation and blade break-off occur at limiting decrease in frequency [23]. The change of the blade frequency also results from erosion and the corrosion. Erosion is reducing mass of the blade and increasing the frequency. The corrosion is reducing the stiffness of the blade, and in the end the frequency of the blade is decreasing. Single compact FOD practically is not changing modal parameters of the blade, but it is a mechanical notch with concentrator of stresses.

A report is describing the diagnostic rule

(2) if  fblade∈〈fmin⁡,fmax⁡〉,then  OKelse  if  fblade>fmax⁡  then   Work  hardening  or  Erosion else  if  fblade≥(fmin⁡−Δfmax⁡)  then       Softening else cracking.

This approach has advantages and disadvantages.

*Advantages*

- Practicable methods in the overhaul and service of the aero-engines;
- Possible active control of blades fatigue;
- Possible comprehensive diagnostics of the engine. The blades of the compressor palisade are mechanical filter about known average parameters.

*Disadvantages*

**Figure 10:**
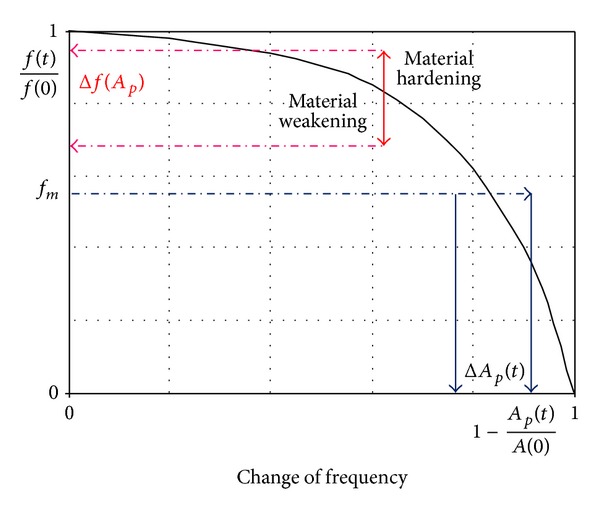
Lack of explicit copying of the size of the crack in the 1st modal frequency of blades [3].

- The frequency value of cracking blades depends on the crack position, parameters at the top of the crack edge (hardening or softening), and the loading. The modal frequency of the blade can suit different sizes of cracks (Figure 10).
- The blade frequency and mass distribution are tuned during assembly. Diagnostic symptom can be distorted during the manufacture and repair of compressor.
- Blades' frequency check offers too short prognosis horizon (Figure 5). It is sufficient in the blade health monitoring only; for example, in the tip-timing method which is used to detect dangerous blades vibration and open cracks during engine operation.

### 3.3. Damage Monitoring of the 2nd Stage

More sensitive detection of fatigue is based on the relationship between changes in the material structure during 1st and 2nd stage of damage (before open crack), Figure 5, and subtle measurable changes of parameters (diagnostic symptoms).

Generally speaking, crystal lattice imperfections have a mechanical strengthening effect, since the lattice defects act as obstacles to the movement of dislocations when a mechanical stress is applied. Different strengthening mechanisms can be distinguished depending on the type of lattice defect contributing to the obstruction of moving dislocations [56, 64, 65]:

- solid-solution strengthening (interstitial/substitu-tional impurity atoms);
- strengthening from point defects (due to vacancies);
- work-hardening or strain-hardening (due to other dislocations);
- grain boundary strengthening;
- martensite strengthening (phase transformation);
- strengthening from fine particles (due to precipi-tates/inclusions).

Compressor blades work in temperature which is below 30% of melting temperature, that is, in *cold working regime*. Overload, stress concentration near nodal line, erosion and corrosion pitting, and fatigue, Table 2, mainly influence on the dislocation density *ρ*_*d*_ and the grain diameter *d*, defined as the average grain diameter. Cold working and fatigue change mechanical parameters of material (yield point *σ*_*y*_, tensile strength *σ*_utl_, hardness and microhardness, Young modulus, damping, and nonlinearity) [66–68]. In the end modal properties of the blade are changing.

The fatigue damage progression can be divided into different (partially overlapping) stages, based on studies of the basic structural changes [56, 64–71].

*Fatigue Damage Initiation*. Even at cyclic stress amplitudes below the macroscopic yield stress, the cyclic mechanical loading can plastically deform the material locally on a microscopically small scale: such microplastic flow first occurs in the grains that are stressed with the highest shear stress amplitude and near inherent material imperfections (inclusions, scratches, and voids). Indeed, tensile residual stress concentrations, associated with such imperfections, lower the actual applied stress at which the material starts to plastically deform locally in some individual grains. The initiation of damage due to cyclic deformation therefore consists of microstructural changes associated with localized micro-plastic deformation in some individual grains, that is, the development of slip bands, the generation of dislocations (increase of dislocation density), and the rearrangement of dislocations into dislocation tangles, dislocation walls, and persistent slip bands. These persistent slip bands can be envisaged as embryonic fatigue cracks.

*Result.* Nucleation of microsized cracks along the developed slip bands in a number of grains.

*Slip Band (Stage-I) Crack Growth*. Consider a microcrack that is initiated inside an individual grain of a polycrystalline material. Such microcrack can grow further under sufficiently applied cyclic stress, along slip planes of high shear stress. Then, to develop further, the crack must propagate into the neighbouring grains, which have different lattice orientations and therefore different slip systems. For small microcracks to propagate, the crack needs to reorientate at the grain boundary towards a particular slip direction of the surrounding grain. Typically, the majority of lifetime corresponds with microcrack (nucleation and growth), which is moreover a regime of stable damage progression. 

*Result*. Formation of dominant crack(s) (with dimensions of typically a few to ten grain diameters wide).

*Transgranular (Stage-II) Crack Growth*. As the microcrack propagates, the plastic zone around the crack tip increases and the resistance to crack growth diminishes. The crack becomes insensitive to grain boundary obstacles and to the particular slip systems of individual grains: the crack now develops in the plane normal to the tensile stress direction, and at much faster rates per loading cycle compared to stage-I crack growth. 

*Result*. Growth of a well-defined crack, along the plane normal to the applied stress direction, and coalescence of microcracks towards a macro-crack with such critical macroscopic dimensions, that the remaining cross-sectional area of the material can no longer support the maximum applied load, and the material fails by ultimate fracture during the last stress cycle.

**Figure 11:**
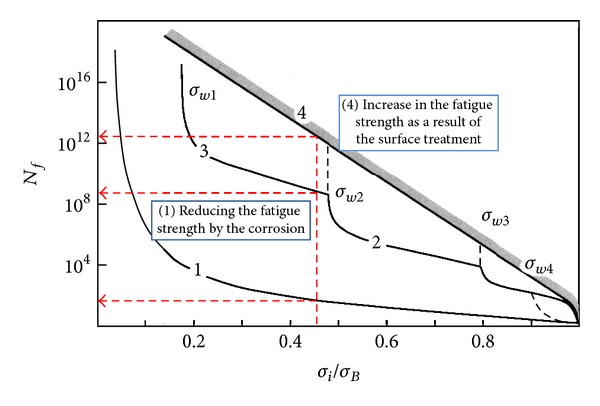
Influence of surface state on the number of cycles to blade failure *N*_*f*_ [3, 56].

The material's performance concerning the fatigue damage process is typically characterized by a *S-N curve*, also known as a Wöhler curve, which gives the cyclic stress amplitude *σ*_*a*_ as a function of the number of cycles to failure *N*_*f*_, the latter in a logarithmic scale. Classically the S-N curve is not crossing 2 · 10^8^ cycles what is insufficient for the compressor blades which TBO is copied through 10^10^ to 10^14^ cycles. Taking the VHCF risk into account requires using the widened S-N curve, appointed on the base of the bimodal theory of the metal fatigue (containing other bifurcations). About the real permanence of the blade (for the given level of exploitation stresses) a state of its surface (level of erosion and/or corrosion, FOD) is deciding (Figure 11).

Strengthening of a metal, which represents the increase in the resistance to yielding or plastic deformation, can be obtained by changes in microstructure that impede the motion of dislocations [67, 68, 71]. Based on the type of obstacles that hinder the motion of dislocation and hence increase the strength, the yield strength *σ*_*y*_ of steels is usually expressed in the form of generalised equation where the contribution of all the strengthening mechanisms is added as follows [72]:

(3) $$\begin{array}{c} \sigma_{y}=\sigma_{0}+\sigma_{\text{SS}}+\sigma_{P}+\sigma_{\text{GB}}+\sigma_{D}+\sigma_{T}, \end{array}$$

where *σ*_0_ is the lattice friction, *σ*_SS_ is the solid solution strengthening, *σ*_*P*_ is the precipitation strengthening, *σ*_GB_ is the grain boundary strengthening, *σ*_*D*_ is the dislocation strengthening, and *σ*_*T*_ is the texture strengthening. 

In theoretical considerations, the Hall-Petch and the Bailey-Hirsch relations (4) between microstructure and mechanical parameters are given as follows:

(4) $$\begin{array}{c} \sigma_{y}≅\sigma_{f}\left(T\right)+\frac{k_{\text{HP}}}{\sqrt{d}},\\ \sigma_{y}\left(\text{after}\;\varepsilon_{p}\right)=\sigma_{0}+\tau_{i}≅\sigma_{0}+\alpha Gb\sqrt{\rho_{d}} \end{array}$$

with *σ*_*f*_ being the friction Peierls-Nabarro stress required to move a dislocation in a single crystal, *T* being material temperature, *k*_HP_ being material-dependent Hall-Petch constant which represents the difficulty required to unlock or generate dislocations in neighbouring grains, *d* being the grain diameter, *σ*_0_ being the lattice friction, *τ*_*i*_ being the shear internal stress, *α* being a constant, *G* being the shear modulus, *b* being the crystal lattice parameter (base length of cubic unit cell), and *ρ*_*d*_ being the dislocation density. 

Typical values for carbon steels are *α*≅0,4, *b* = 0.286 nm, *d* = 10^−4^–10^−6^ m, *G* = 80 GPa, $k_{\text{HP}}=0.74\;\text{MPa}\sqrt{m}$, *σ*_*f*_ = 70 MPa, and *σ*_0_ = 100 MPa.

The residual “life” of material *ζ* (relative residual time to blade break-off) is given by local level of dislocation density and the following relation:

(5) $$\begin{array}{c} \zeta =1-\frac{\sqrt{\rho_{d}}-\sqrt{\rho_{d0}}}{\sqrt{\rho_{d\max}}-\sqrt{\rho_{d0}}} \end{array}$$

with *ρ*_*d*0_ being the dislocation density for well-annealed materials, and *ρ*_*d*max⁡_ being the dislocation density for ductile strength. Typical values for carbon steels are *ρ*_*d*0_ = 10^−10^ m^−2^, *ρ*_*d*max⁡_≅10^−15^ m^−2^.

The second stage of blade damage can be observed, for example, in “a resonance curve,” (RC) measured by a laser point head during modal frequency testing [3, 23]. This approach is sufficient to solve overhaul problem (level II) with experimental modal analysis method. A report is describing the diagnostic rule (6), which will be developed at the description of method:

(6) if RC is for a linear object, then  OK or Work hardening   else Softening or Cracking.

This approach has advantages and disadvantages.

*Advantages*

- Simple algorithms of the data analysis and diagnostic rules.
- High performance of examinations average time of objective testing (of parameters chosen mod and of the structural health condition) does not exceed 3 minutes per the blade.
- Possible reliable prognosis and reduce of the risk of wrong diagnosis during engine overhaul.
- The method is made available with base knowledge about relations between the material fatigue and modal properties which is being used by the tip timing method.

*Disadvantages*

- Required is direct access to the blades.
- The approach requires the selection of a new sensitive observers (measurement technics and analysis methods) as well as recognition and verification of new diagnostic symptoms.

### 3.4. Damage Monitoring of the 1st Stage

**Figure 12:**
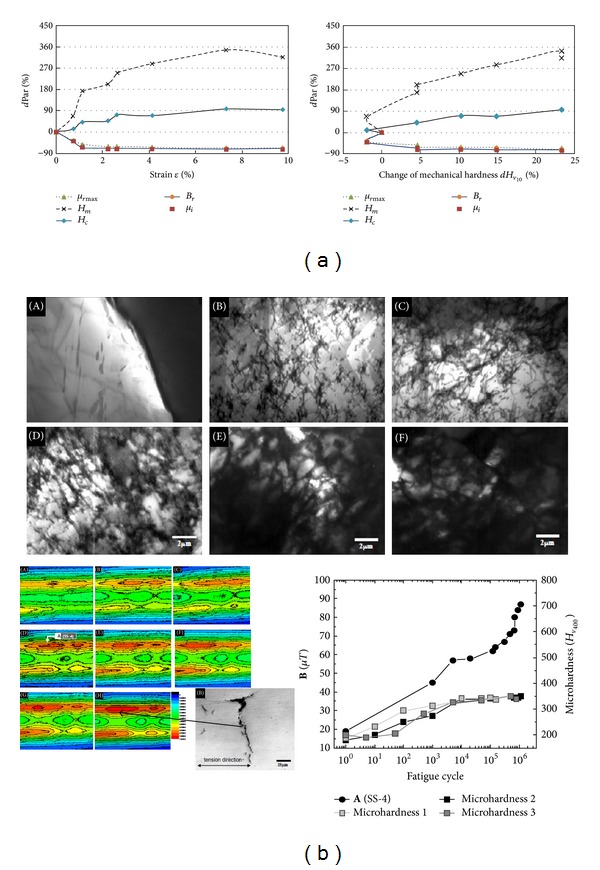
Showing (a) influence of static strain hardening on magnetic parameters low carbon steel with 0,17% C (*H*_*m*_ = *H*(*μ*_*r*max⁡_)),  *d*Par = (Par(*ε*) − Par(*ε* = 0)/Par(*ε* = 0)) [73]; (b) influence of HCF on TEM microstructure of fatigue 430 stainless steel (after 0 cycles, 1 × 10^3^ cycles, 1 × 10^4^ cycles, 2 × 10^4^ cycles, 4 × 10^5^ cycles, 5 × 10^5^ cycles) and remanence magnetization map of the steel (after: (A) 0 cycles, (B) 1 × 10^3^ cycles; (C) 5 × 10^3^ cycles; (D) 2 × 10^4^ cycles; (E) 1,2 × 10^5^ cycles; (F) 1,45 × 10^5^ cycles; (G) 2,8 × 10^5^ cycles; (H) 9,2 × 10^4^ cycles. SQUID scanning area 10 × 20 mm) [74].

If the blades are made of ferromagnetic material, the biggest extension of the prognosis horizon is possible. The dislocation density, the diameter of the grain, the history of mechanical load (residual stress) and applied loads change not only mechanical parameters (3) but also the state of magnetizing blades, see Figure 12 and relation (7). Changes of material magnetizing, resulting from lattice-spin coupling (L-S) in the atomic scale, distribution of residual stress in the atomic micro- and macroscale, and magnetomechanical effects (reversible and irreversible) in the macroscopic scale, enable the detection of 1st phase of the damage [73–75]:

(7) M=Mi+Mr=(1+kH)(1+kσ)(1+kT)M0Hc∝{ρd,d−1}→{Li−1,σr}Br,μrmax⁡∝{ρd−1,d}μi∝{Li2,σr−1}

with **M** being magnetization; **M**_*i*_ being the induction magnetisation; **M**_*r*_ being the residual magnetisation; **M**_0_ being initial state of magnetizing the blade; *k*_*H*_, *k*_*σ*_, *k*_*T*_ being appropriately influence of external field, stress, and temperature; *H*_*c*_ being the coercive force; *B*_*r*_ being the remanence; *μ*_*r*max⁡_ being the maximum magnetic permeability; *μ*_*i*_ being the initial magnetic permeability; *σ*_*r*_ being the magnitude of unidirectional internal stress which represents the irregularly fluctuating magnetoelastic energy distribution; *L*_*i*_ being the periodic distance between internal stress centre [76].

Magnetic properties of the blades are dependent on the microstructural type, of additions alloy and residual stresses, as well as level of material damage. A report is describing the diagnostic rule (8), which will be developed at the description of method:

(8) if  B∈〈Bmin⁡,Bmax⁡〉, |dBdx|<|dBdx|max⁡then  OKelse  Hardening,Erosion,Softening,Cracking,Stress  Concentration  Zone.

This approach has advantages and disadvantages.

*Advantages*

- Detection of reversible changes of the material fatigue (1st phase of damage).
- Possible of remote observation of magnetizing blades through paramagnetic casing of compressor. It is sufficient to solve service problem—detect fatigue risk before the first blade crack (level I).
- Observation of magnetizing blades during the engine stop (using irreversible magnetomechanical effects), the small rotation speed (from zero rpm), and the engine work on the operation range.
- Possible reliable prognosis and reduce of the risk of wrong diagnosis during engine overhaul and service.
- Possible analysis postfactum of damage elements and identification of load condition prevailing during the initiation and the propagation of the crack.
- The solution can be used for diagnosing other ferromagnetic elements of the plane, for example, of bearing, gears, shafts, pressure vessels, and landing gear.
- The method is made available with base knowledge about relations between the material fatigue and stress-strain inducted magnetization and symptoms using by the experimental modal analysis and the tip timing methods.

*Disadvantages*

- Strong nonlinear rules describing the magnetization of the ferromagnetic parts, particularly in the weak magnetic field.
- The approach requires the selection of a new sensitive observers (measurement technics and analysis methods) as well as recongnition and verification of new diagnostic symptoms.
- Only for ferromagnetic materials and some paramagnetic steel.

## 4. SHM and NDE Methods

Magnetic and magnetomechanical properties of ferromagnetic blades are changing their mechanical and modal properties. The state of magnetizing blades also affects the tip timing signal (when there is an induction, vary reluctance or eddy current sensor is used to detect moving blades). The theory and experience of experimental modal analysis are an input to the tip timing method. For paramagnetic blades which material is not showing of phase transformation under the influence of stresses, the first method (Metal Magnetic Memory method) is not applicable. 

### 4.1. The Metal Magnetic Memory Method

The MMM method (only NDE method according to ISO 24497:1-3 (2007)) is based on three pillars:

- magnetomechanical effects existing in ferro-magnetic material being located in a weak magnetic field of the Earth which was described by theory of the micromagnetism [76–88];
- magnetovision: the remote passive observation magnetic field near the testing element;
- magnetostatic: solving the opposite issue of magnetostatic in the destination of the magnetizing trend from magnetic anomaly (symptom of defects, stress concentration zone, and change of element shape).

**Figure 13:**
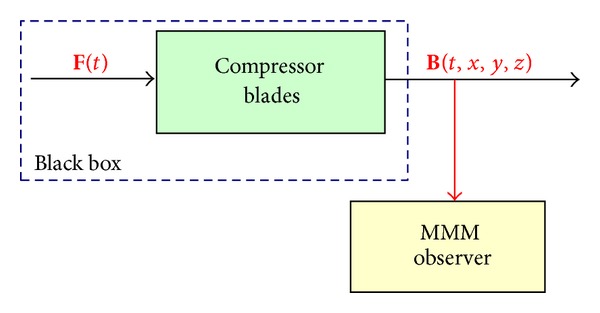
Signal analysis of the blade magnetizing (magnetic induction **B** in the close of the its surface) which the health monitoring and load history of the blade described.

The MMM method is a typical passive observer of *the signal analysis* which is possible to apply both to NDE and SHM applications. Signal analysis is the process of determining the response of a system, due to some generally unknown excitation, and of presenting it in a manner which is easy to interpret (Figure 13).

In the macroscopic scale a constitutive law (9) is copying magnetic properties of the blade:

(9) $$\begin{array}{c} B=\mu H=\mu_{0}\left(H+M\right) \end{array}$$

with *μ*_0_ being magnetic permeability of vacuum (in SI units system *μ*_0_ = 4*π* · 10^−7^ H/m); **M** being material magnetization [A/m], **H** being external magnetic field [A/m], and **B** being magnetic induction (magnetic flux density) [T].

#### 4.1.1. Reversible Magnetomechanical Effects

**Figure 14:**
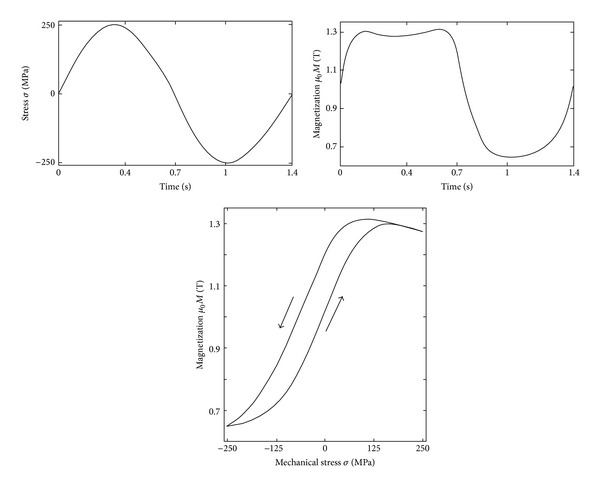
Stress inducted magnetization in low carbon steel (*R*_*e*_ = 390 MPa, stress level below yield strength, static magnetic field *H* = 800 A/m) [76].

Apart from the constitutive law **B**(**H**) or **M**(**H**), there is a second class of macroscopic observations that needs to be introduced. It is observed that when a ferromagnetic specimen is subjected to a magnetic field, its magnetization as well as its length change—Joule effect, which is described by tensor rule (10). When a ferromagnetic specimen is subjected to a mechanical stress, both it is length as well as it is magnetization change—Villari effect, which is described by tensor rule (11) and depicted in Figure 14. The actual distribution of material magnetization (molecular currents in the material) can be observed indirectly by measuring the magnetic field distribution in the nearby the object.

(10) $$\begin{array}{c} \varepsilon_{ij}=s_{ijkl}^{HT}\sigma_{kl}+d_{ijn}^{}H_{n}, \end{array}$$

(11) $$\begin{array}{c} B_{m}=d_{mij}^{*}\sigma_{ij}+\mu_{mn}^{T\sigma }H_{n}, \end{array}$$

where *d* = ∂*ε*/∂*H*|_*σ*_ and d* = ∂*B*/∂*σ*|_*H*_ are magnetomechanical coefficients which are appointed experimentally for given material at the constant tensile (stresses) *σ* or constant magnetic field *H* [75].

In (10) and (11) an influence of the change of material temperature and losses of the internal energy were omitted. Equations are describing only “reversible magnetomechanical effects.”

Zones RSC of local residual stress concentration, plastic, material anisotropy (mechanical and magnetic) and dislocation concentration are potential place of cracking nucleation and local magnetic anomaly [4, 72, 76–79]. Influence of the local plastic strain of material (LCF, HCF and VHCF problems—Table 2) the best is visible in the weak magnetic field [80]. “The passive magnetic observer of the blade health monitoring (e.g., the metal magnetic memory method) is favoured.”

#### 4.1.2. Magnetomechanical Damping

Applying a stress to a ferromagnetic blade causes a variation of magnetization due to the magnetoelastic coupling, which results in the so-called “Δ*E* effect” and also in a related dissipation of mechanical energy during loading/unloading or in case of vibration. The latter effect can give rise to a strong magnetomechanical damping with stress-dependent and stress-independent components [81].

Experiments show that ferromagnetic materials have a higher internal friction than the paramagnetic and diamagnetic because of phenomena of an electromagnetic nature resulting from the application of elastic fields. Considering five main contributions to the total energy of a ferromagnetic material without an external field (exchange energy *W*_ex_, magnetocrystalline anisotropy energy *W*_*k*_, magnetoelastic (or magnetostrictive) energy *W*_*λ*_, magnetostatic energy *W*_*m*_, and energy of magnetic domain walls *W*_*w*_), four main mechanisms of magnetomechanical damping may be defined:

- magnetoelastic hysteresis damping *Q*_*h*_^−1^,
- macroeddy-current damping *Q*_*a*_^−1^,
- microeddy-current damping *Q*_*u*_^−1^,
- damping at magnetic transformation *Q*_*PhT*_^−1^.

Therefore, the total magnetomechanical damping *Q*_*m*_^−1^ in ferromagnetic blade can be considered as sum of these components:

(12) Qm−1(ε,ω,T)=Qh−1(ε,ω,T)+Qa−1(ω,T)+Qμ−1(ω,T)+QPhT−1

contrary to *Q*_*a*_^−1^ and *Q*_*u*_^−1^, the hysteretic contribution *Q*_*h*_^−1^ depends on the strain amplitude. The damping *Q*_*m*_^−1^ is also dependent on the load frequency *ω*, material temperature, and initial conditions (micro- and macrostructure, magnetization, and residual stress). “The *Q*_*m*_^−1^ is nonlinear.”

#### 4.1.3. Irreversible Magnetomechanical Effects

Losses of the internal energy are being observed in the weak DC magnetic field in the form of

**Figure 15:**
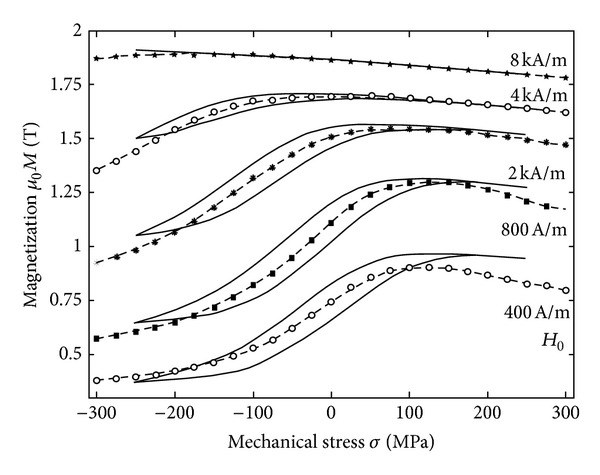
Experimentally obtained magnetomechanical hysteresis loop *M*(*σ*(*t*)) for different setting of external DC magnetic field *H*_0_ (full lines) and compared to the reversible anhysteretic magnetomechanical behaviour at corresponding setting of *H*_0_ (dashed line with symbols). Material: low carbon steel with 0,12% C [76].

- the magnetization hysteresis loop (Figure 15);
- growing magnetizing material under the influence of the cyclical load (LCF an HCF fatigue) [82]. Observed change of magnetizing material depends on the level of stresses and the number of cycles;
- change of magnetizing material after unloaded (“the metal magnetic memory” or “the first loading/unloading effect”) [77, 83]. The ferromagnetic blade has feature of the strain gauge with the memory of the maximum load.

#### 4.1.4. Measuring Equipment

Potential possibilities of the MMM method were tested in active and passive experiments, which used a compass (simple magnetometer), GM-04 Magnaflux magnetometer with Hall sensor [89], 3D MEMS anisotropic magnetoresistive sensor (HMC 5843 Honeywell demo board) [90], and Energodiagnostika TSC-1 M-4 recorder with multichannel transduction sensors (scanning devices) [91]. The Earth's magnetic field (*B*_*E*_≅50 *μ*T) and electromagnetic noise are natural source of the external magnetic field.

### 4.2. The Experimental Modal Analysis Method

Experimental modal analysis (a tool of structural analysis) is an effective aid in solving blades' fatigue problems. It allows finding an answer to the question: “Why does a blade crack?”, not only: “Is it cracked?”. The modal parameters of all the analysis modes (within the frequency range of interest) constitute a complete dynamic description of the blade structure [3, 17–21]:

- material,
-  geometry,
- the influence of surface treatment and adding protection coating,
- technical health (structural heterogeneity, crack, and fatigue).

**Figure 16:**
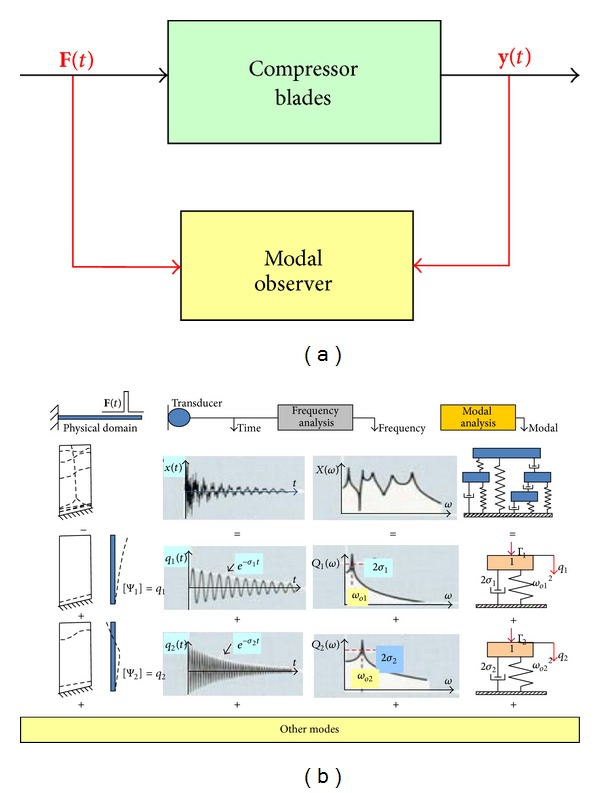
Blade health and mechanical properties analysis using the experimental modal analysis.

The characteristic feature of blade vibration measurement on a modal excitation system is knowledge of both a force level and a blade response on it (Figure 16(a)). This can be done by stimulating the system with *measurable force* and studying *the response*/*force ratio*. For linear system this ratio is an independent, inherent property which remains the same whether the system is excited or at rest. That is why it is possible to identify blade modal properties for following modes. 

Structural response of the compressor blade (*a lightly damped structure*) can be represented in different domains. The modal description relates to descriptions in the spatial, time, and frequency domain (Figure 16(b)).

In the illustration, each column shows the response of the blade after short struck represented in different domains.

*Physical domain*: the complex geometrical deflection pattern of the blade can be represented by a set of simpler, independent deflection patterns, or mode shapes.

*Time domain*: the vibration response of the blade is shown as a time history, which can be represented by a set of a decaying sinusoids.

*Frequency domain*: analysis of the time signal gives us a spectrum containing a series of peaks, shown below as a set of SDOF (single-degree-of freedom) response spectra. 

*Modal domain*: we see the response of the blade as modal model constructed from a set of SDOF models. Since a mode shape is pattern of movement for all the points on the structure at a modal frequency, a single model coordinate *q* can be used to represent the entire movement contribution of each mode. The SDOF model is associated with a *frequency*, a *clamping*, and a *mode shape*. An important property of modes is that any forced or free dynamics response of structure can be reduced to a discrete set of modes. The modal parameters are as follows:

- modal frequency;
- modal damping;
- mode shape.

#### 4.2.1. Measuring Equipment

**Figure 17:**
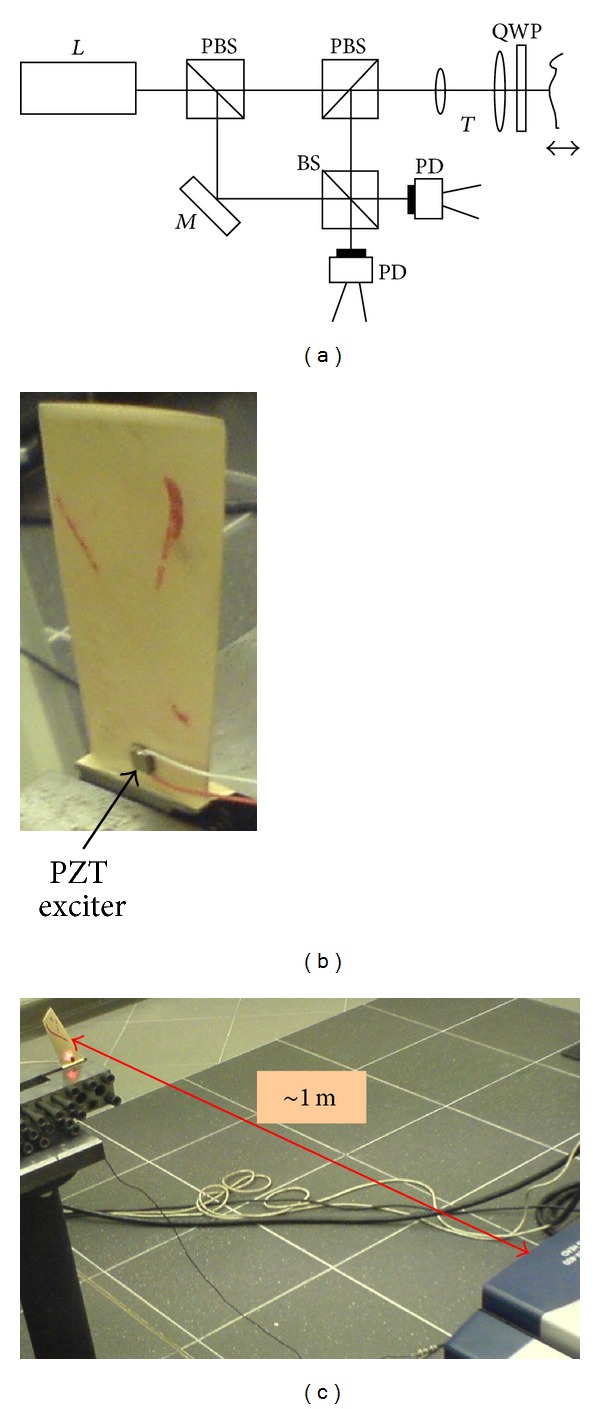
Experimental stand for the broadband (up to 20 kHz) modal identification of the compressor blade with the use of scanning laser vibrometer and PZT exciter.

The broadband identification (up to 20 kHz) of modal properties of a compressor blade, made of 18H2N4WA steel and Ti5.8Al-3.7Mo titanium alloy, has been made on the PSV-400 Polytec scanning vibrometer [92] and low power PZT exciter (Figure 17).

This approach has advantages and disadvantages.

*Advantages*

- Automation of the measurement;
- Mode frequency and node lines are very precisely identified;
- Weak clamping of blade is possible;
- Input data to numerical modeling of modal and fatigue properties;
- Vibration frequency up to 20 MHz and vibration velocities up to 20 m/s;
- Scan area (±20° about *X*, *Y*) and grid definition;
- High speed (>50 points/s) and resolution (<nm, 0.002°);
- High angular stability (<0.01°/h).

*Disadvantages*

- High cost and weight measurement equipment (7,5 kg);
- The small vibration amplitude is not opening the crack.

**Figure 18:**
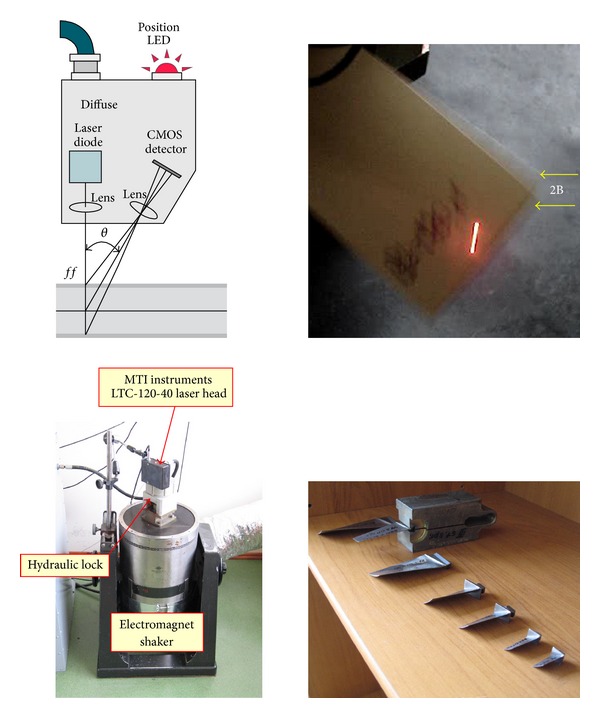
Workstation for the low-frequency (up to 4 kHz) modal identification of the compressor blades.

The identification of early fatigue and cracking symptoms of these blades has been made on the Brül & Kjær electro-dynamic exciter 4802T [93]. The experimental stand (Figure 18), used during the SO-3 and TW3-117 engine overhauls, included the following:

- the MTI Instrument laser measurement system MicroTrack II with CMOS measurement head LTC-120-40 [94];
- the Vibration Research Corporation VR-8500 controller that includes 24 bit A/D and D/A converters, and RISC processor [95];
- the Vibration Research Corporation Vibration View software to control the exciter, data acquisition, and analysis [96].

The sensitivity of measurement system is 100 mV/mm.

This approach has advantages and disadvantages.

*Advantages*

- Load up to 60 g (*a* = 588 m/s^2^)—LCF & HCF test is possible;
- Low cost of laser head;
- Frequency response: 20 kHz max (laser) and 4 kHz max (exciter);
- Resolution at 20 kHz filter: ±5 *μ*m;
- Filter setting: 20 kHz–0.1 Hz;
- High temperature stability (0.005%/K) and linearity (0.05% FSR or better).

*Disadvantages*

- Single point measurement;
- Great demand of the electric power.

### 4.3. The Tip Timing Method

**Figure 19:**
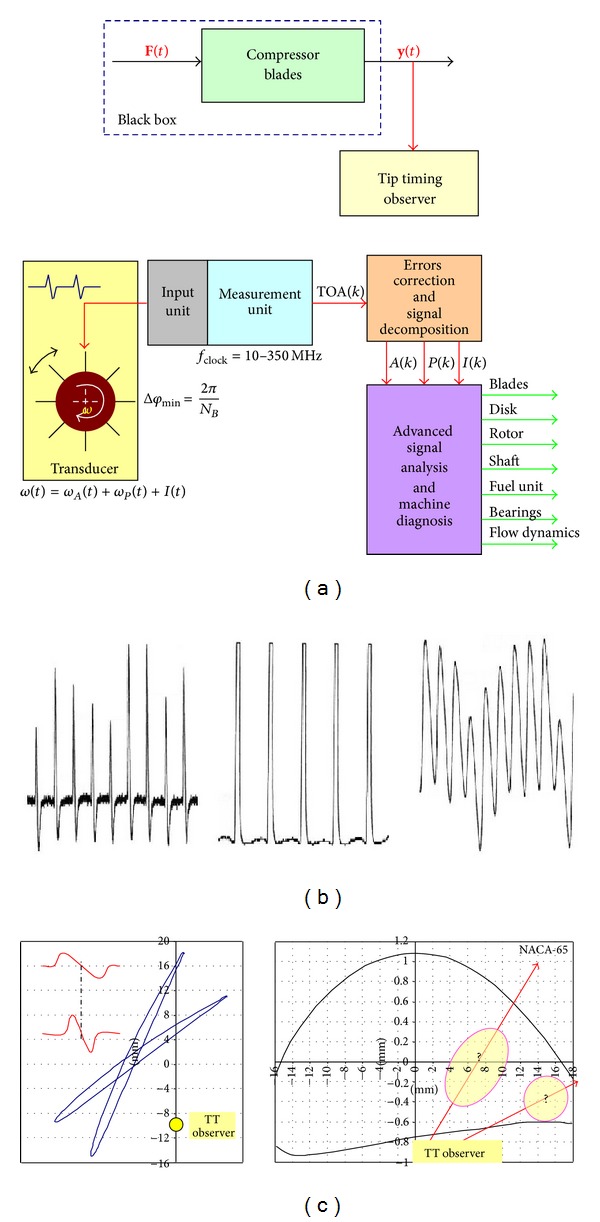
Showing (a) an idea and a block structure of the tip timing method; (b) a shape of analog signal for vary reluctance (VR), optical and eddy current (EC) sensor. Signal with VR and EC sensors also contains information about magnetizing blades; (c) main problem of VR and EC sensors—precise relation of the analog signal with putting the top of the blade with regard to the sensor (Where is the blade? Which point of the blade top is in the relation with the characteristic point of the analog signal?) [25].

The tip timing idea consists in observing displacement of loaded component part, Table 3, with “irregular sampling” (one time to the turnover of the rotor). In our case, it will be a rotating and vibrating compressor blades. Blade vibration and deflection are a source of a time-interval change between flexible key phases. The tip timing observer (sensor) is built onto a fixed part of compressor. Its analog signal depends on the sensor type (Figure 19). Time period signal (time of blade arrival, TOA) would be measured with a frequency method or delay line (time-to digital converter) [3, 25, 96, 97]. 

Measured signal TOA(*k*) is discreet representatives of the continuous signal *S*(*t*) which contains the following:

- aperiodic part *A*(*t*)—average instantaneous rotational speed of perfect stiff rotor;
- oscillating part *P*(*t*)—resultant from pitch errors, blade, rotor and disk vibration, and instantaneous rotational speed perturbations (from the engine control system, flow, g-force, clearance in a kinematic system, and torsional vibration);
- noise and weak oscillating components *I*(*t*).

Signal *S* is described by the following relation:

(13) $$\begin{array}{c} S\left(t\right)=A\left(t\right)+P\left(t\right)+I\left(t\right) \end{array}$$

so it is possible to design a general-purpose observer for *real operating conditions* of rotating parts and have a complex view on the following:

- disadvantageous dynamic phenomena (flutter, stall, surge, resonance, and load coupling);
- the influence of production, overhaul, and maintenance real conditions on the level of malfunctioning and fatigue prognosis.

Every component of *S*(*t*) is used to diagnose. An oscillating part *P*(*t*) is a main carrier of diagnostic information about blades damage and danger dynamics phenomena. Aperiodic part *A*(*t*) and part *I*(*t*) give the capability to compare new diagnostic symptoms to the health of machinery.

Signal TOA(*k*)—a number of pulses Code_*i*_ with clock frequency counting between key phases (blades)—includes “three groups of variables” (14) to be identified in effect of further numerical signal analysis

(14) TOA(k)=Codei=Ki,i+1Trunc(ti,i+1tclock)=(1+ζB1+ζωTOAavg)i,i+1, i∈〈1,2,…,NB〉,TOAavg=2π/NBω

with *k* being discrete time, *K*_*i*,*i*+1_ the error and disturbance factor (*K*_*i*,*i*+1_ = 1 for data without error), *N*_*B*_ the number of blades, *t*_*i*,*i*+1_ the time interval between two blade passes (with momentary pitch), *t*_clock_ the time period of generator pulses (with model of the time), TOA_avg_ the average momentary time of arrival of the perfect rotor and the blade palisade (without influence of rotor unbalance and vibration and blade pitch errors), *ζ*_*B*_ the jitter of blades group components, *ζ*_*ω*_ the jitter of rotor group components, and *ω* the angular velocity of ideal rotor.

The jitter of blade group components includes

 
*ζ*_*P*_: pitch errors (*N*_*B*_ of aperiodic variables);
 
*ζ*_*Bi*_: vibration of *i* the blade (*N*_*B*_ of independent multimodal generators);
 
*ζ*_*C*_: compressor case vibration;
 
*ζ*_DP_: dynamic phenomena of TTM sensor

so it is described by the following relation:

(15) $$\begin{array}{c} \zeta_{B}\left(k\right)=\zeta_{P}\left(k\right)+\zeta_{Bi}\left(k\right)+\zeta_{K}\left(k\right)+\zeta_{\text{DP}}\left(k\right). \end{array}$$

The jitter of rotor group components includes

 
*ζ*_*F*_: influence of control unit and changes of the momentary rotation speed;
 
*ζ*_*R*_: transverse and torsional vibration of rotor;
 
*ζ*_*E*_: alignment error (eccentricity)
 
*ζ*_*A*_: alignment error (misalignment),

so it is described by the following relation:

(16) $$\begin{array}{c} \zeta_{\omega }\left(k\right)=\zeta_{F}\left(k\right)+\zeta_{R}\left(k\right)+\zeta_{E}\left(k\right)+\zeta_{A}\left(k\right). \end{array}$$

**Figure 20:**
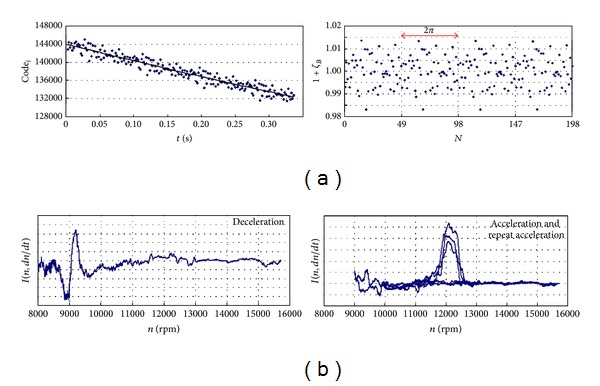
Result of the DETREND procedure for TOA(*k*) signal measured for (a) the last stage of SO-3 engine compressor (49 blades in the palisade)—the pitch errors are dominating in the jitter *ζ*_*B*_; (b) the first stage of SO-3 engine compressor—the component *I*(*k*) = *I*(*n*, *dn*/*dt*) is revealing the influence of some asynchronous parts of jitter *ζ*_*ω*_ on the component *A*(*k*) (e.g., rotor vibration, fluctuation in the rotational speed on the compressor limits) [3].

The jitter *ζ*_*ω*_ is a source of FM modulation which is mainly problem of TOA(*k*) signal disintegration (to components *A*, *P*, and *I* after signal verification and numerical correction) and analysis of the blades health and vibration. Signal components of TOA(*k*) are obtained with the DETREND procedure (Figure 20).

The scope of interest of numerical data processing includes [3, 25–33]

- vibration level of all blades at the same time,
- disadvantageous dynamic phenomena,
- blade stress and health,
- disk health,
- engine health (the engine fuel system and the bearing system).

**Figure 21:**
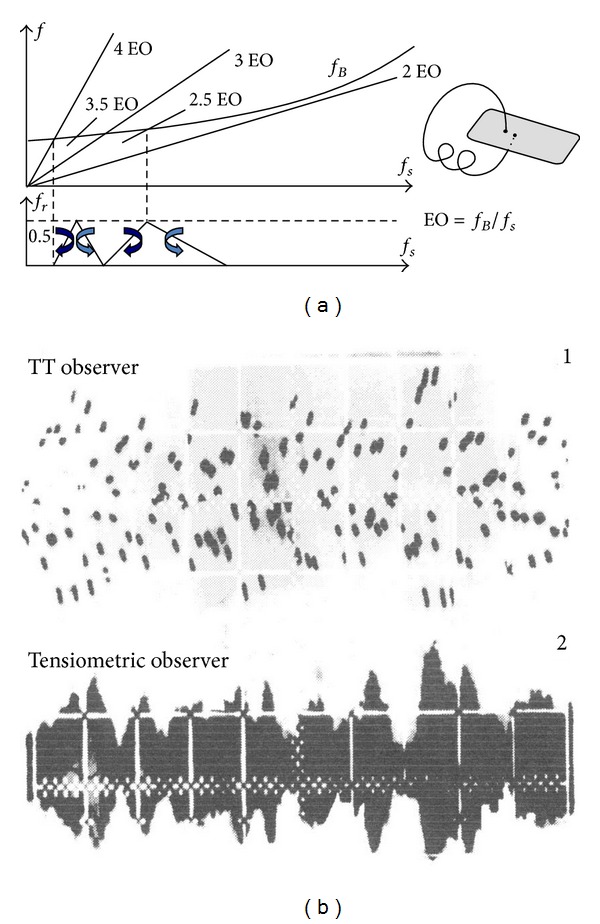
Relation between vibration of the blade top (the TT observer, *f*_sampling_≅*f*_rotor_ ± Δ*f*_jitter_) and stresses at the base of the one (the strain gauge observer, *f*_sampling_=4 kHz). Three first modal frequencies of the blade: 350 Hz, 1380 Hz, and 1890 Hz, max. rotational frequency of the rotor *f*_rotor,max⁡_ = 260 Hz [55].

Signal subcomponents are obtained with the narrow-band filtering, AM/FM demodulation and spectrum analysis (e.g., CORDIC, DFT and DASP algorithms are used). Blade vibrations are shown in the form of phase distributions as points of phase trajectory crossing the phase plane [25, 55] (Figure 21).

A main characteristic feature of the tip timing method is information that lasts about a total number of modal frequency periods between two subsequent points of phase trajectory crossing the phase plane, with basic modal parameters of the blades preserved. This phenomenon enables detection of the LCF and HCF crack initiation and propagation in the blade during the engine operation. Other characteristic features of the TTM are the following.

- Irregular signal sampling rate: the Nyquist-Landau law describes discrete-time information.
- Periodic measurement data structure: data can be illustrated with matrix with *N*_*B*_ columns (number of blades) and rows that represent each full 360 degrees cycle of a rotor.
- The inherent in a signal oscillating parts that are not connected with blade vibration: there are two groups of oscillating parts of a signal: synchronized and nonsynchronized with rotor rotational frequency.

#### 4.3.1. The SNDŁ-1b/SPŁ-2b System

In 1993, a diagnostic system was developed and introduced into the service on the TS-11 “Iskra” trainer. The system is based on results of the active and passive tests. It consists of [62] the following.

- *The blade excessive vibration warning device SNDŁ-1b*: a two-channel analogue phase detector that warns a pilot of conditions that can induce accelerated HCF of blades, for example, deposition of foreign matter such as ice, bird, or other resonance-based phenomena.
- *The ground-based inspection instrument SPŁ-2b (digital phase detector, f*_clock_ = 10 MHz*) for*
  - periodic recording of blade vibration,
  - inspections of the SNDŁ-1b health, with no need to have it disassembled,
  - detection of errors of the engine rotational-speed indicators (in cabins I and II), with no need to disassemble them.
- *The SPŁ-2b software*: a set of programs that form the nucleus of the advisory/expert system used to diagnose the SO-3 engine. The software comprises
  - *the database with text data*: that is, a verified set of information on the object under examination and technical specifications of operating/monitoring it;
  - *the database containing measuring data*: more than 7000 records on the Polish population of the SO-3 engines, collected during more than 20 years, taken at OAT = 248 to 308 K (−25 to +35°C), *p*_*A*_ = 959,9 to 1026,6 hPa (720 to 770 mm Hg), and humidity of 20 to 100%; also, the SO-3 overhaul-delivered data (collected during 15 years), including, among other things, information on frequency spacing of blades in the blade ring. The base also includes hidden software usage files (*logs*). They are made automatically without user knowledge. They are the base to valuate diagnostics system usage correctness;
  - *the database containing numerical models*: compressor blades, the fuel system, and the rotor bearing system. The models are also used to identify how errors made during manufacturing, operation, and repairs/overhauls can affect the engine operational safety. Another application can be the postfactum analyses of air accidents;
  - *the database with diagnostic rules*: contains the algorithms used to interpret measuring results and bringing diagnosis of the first-stage compressor blades and the transmission, as well as the expert diagnostic of a fuel system. The diagnostic rules also facilitate automatic identification and verification of the source of measurement data (the engine type and the serial number thereof) and software-based synchronisation of taking subsequent records. The database comprises also procedures of identification and correction of measurement errors and procedures of identification of errors in the operational use of the diagnostic system.

## 5. Research Results

Chosen findings were obtained during active and passive experiments.

### 5.1. The Metal Magnetic Memory Method

**Figure 22:**
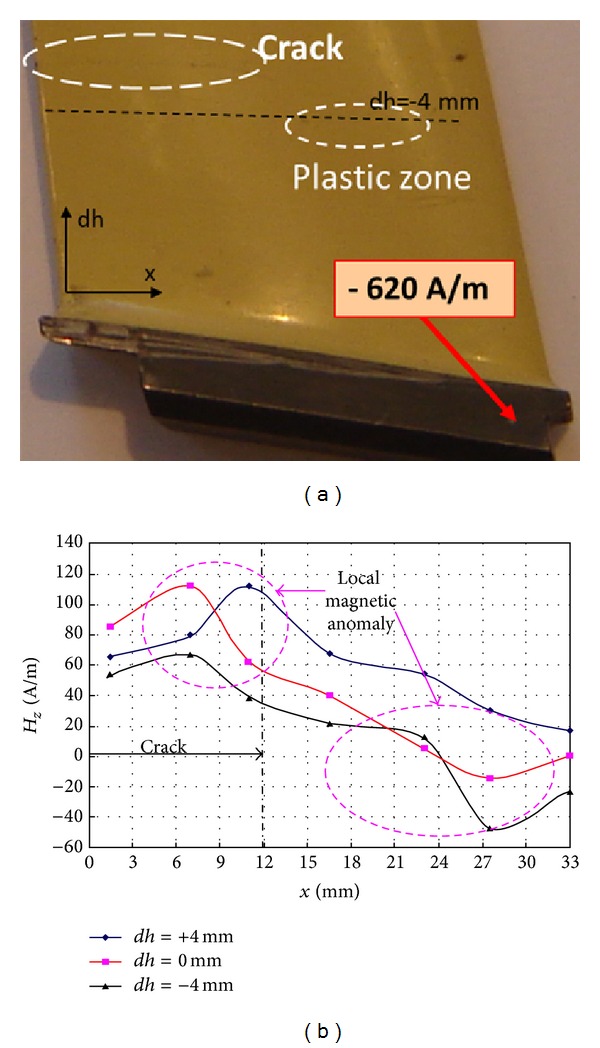
MMM symptoms of cracked blade after HCF test [3, 22].

**Figure 23:**
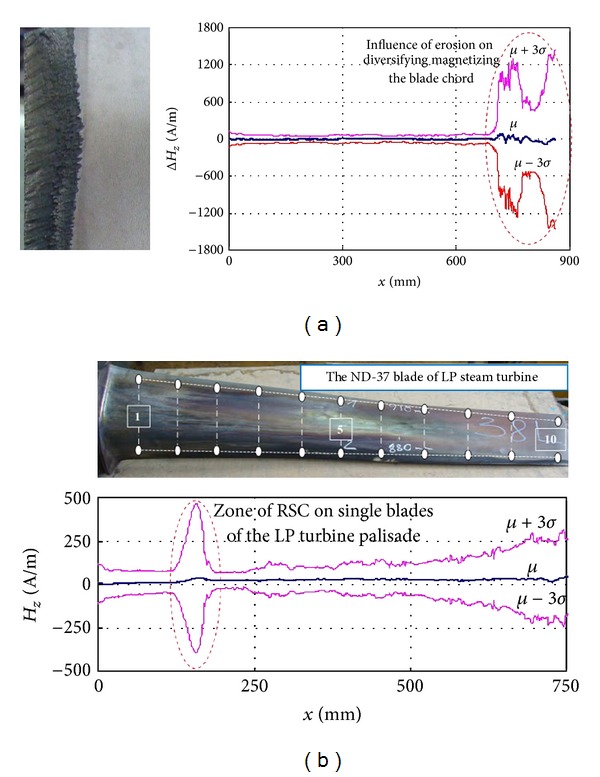
Early detection of the hidden fatigue risk (blade overload and diversifying the income of erosion) on the example of the ND37 blades of the steam turbine [3].

**Figure 24:**
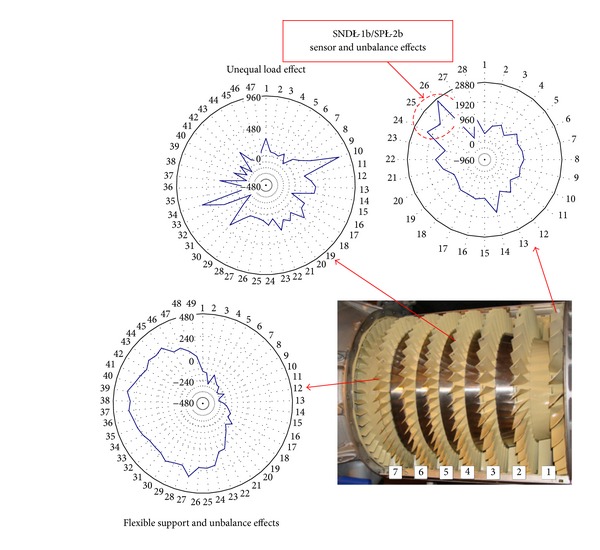
Detection of stress prehistory (irreversible process of stress magnetization) and identification of blade fatigue risk [3, 22].

**Figure 25:**
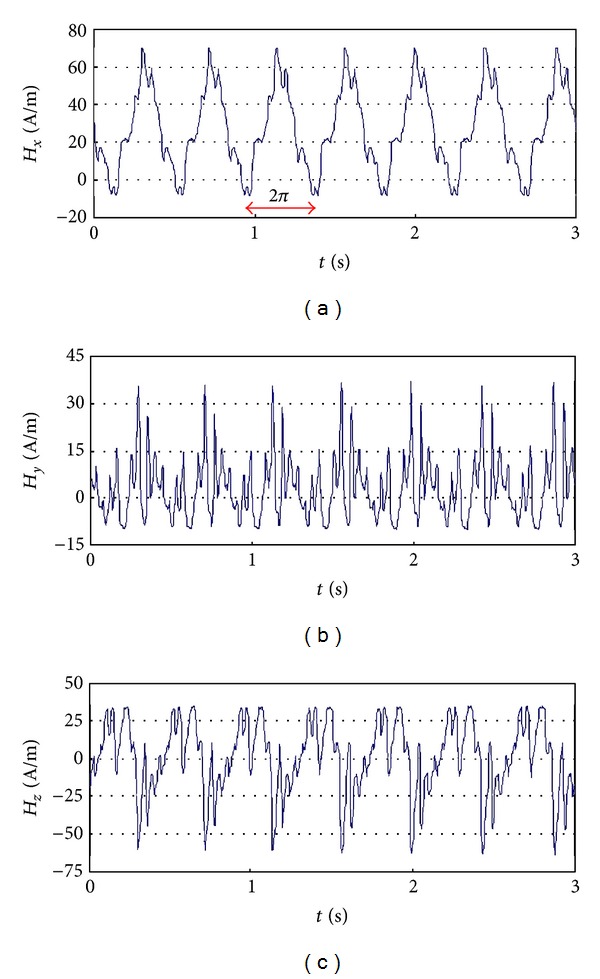
Components of the magnetic field measured on the suface of compressor casing (*H*_*x*_: along the pivot of the engine, *H*_*y*_: tangensial to the casing, and *H*_*z*_: normal to the surface) [3].

Very good relation has been observed between the MMM results and blade node lines after LCF tests [3]. Local magnetic anomaly has been also observed near the close crack gap after HCF tests (Figure 22). The MMM method is widening the possibility of the blade verification by the RSC detection before the appearance of the endurance fracture (Figure 23). Nevertheless, the most interesting phenomenon is nondestructive detect of blade erosion and stress prehistory (a change of residual magnetization) after the engine stopped (Figure 24). Based on previous research and theoretical evidence does not rule out the possibility of diagnosing VHCF problems by MMM method. During measurements of the state of blades magnetizing through the compressor casing new symptoms was demonstrated for the tip timing method (Figure 25).

### 5.2. The Experimental Modal Analysis Method

Experiments have been performed in five stages in which

- the measurement method has been verified,
- blade modal properties have been identified,
- blade cracking symptoms have been identified,
- early symptoms of fatigue have been identified,
- new diagnostic symptoms have been verified for titanium blade.

#### 5.2.1. Identification of the Modal Properties

**Figure 26:**
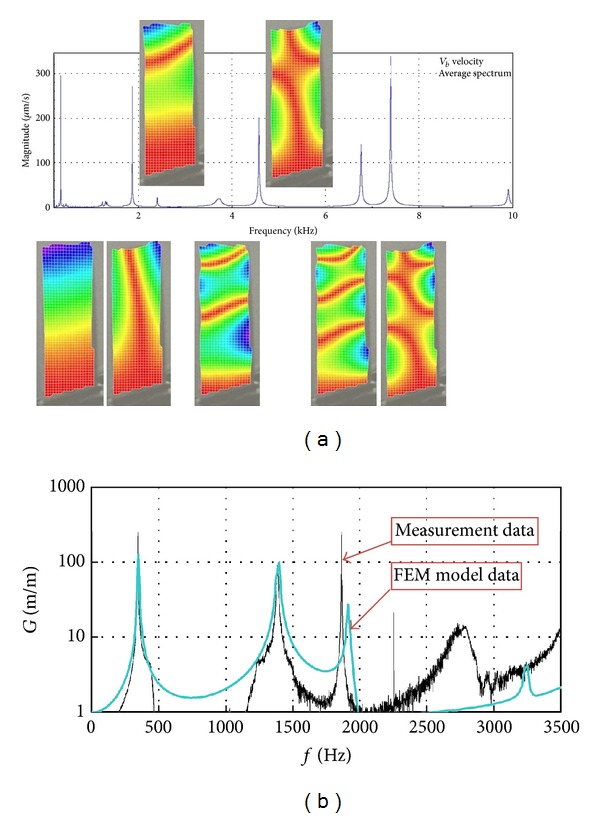
Identification of blade modal properties using (a) PZT exciter and laser scanning vibrometer (906 test points); (b) experimental data and FEM model of the steel blade (before the FEM model is tuning) [3, 22].

It was proven that PZT exciter and Polytec scanning vibrometer could be used for the modal identification of compressor blades. Measuring collected data is a verified knowledge used for tuning the FEM model up (Figure 26).

It has been also proven that used simple measurement technique (MTI laser head and sine test) guarantees reliable modal results when vibration amplitude is higher than 2 *μ*m. Reliable resonance curve shape during sine test has been obtained for force frequency: 2.5 Hz/min for 1st flexible mode (1*F*, *Q*_*s*_ > 350) and 1.0 Hz/min for 1st torsion mode (1*T*, *Q*_*s*_ > 1000). Such a stand gives an ability to make precise measurements with an exact test profile and frequency step. The measurement system gives almost laboratory accuracy. That's why it let [23]:

- precise identification of blade modal properties in measured frequency range;
- metrological factors influence analysis on recorded resonance characteristics;
- modal parameters trends analysis be observed during fatigue tests.

Hereinafter of point 5, results will be presented from sine test. To analyze data we can use operator transmittance described by the following relation

(17) $$\begin{array}{c} G\left(\omega \right)=\frac{Y\left(\omega \right)}{X\left(\omega \right)}\left[\frac{m}{m}\right] \end{array}$$

with *X*(*ω*) being the magnitude of the exciter head displacement; *Y*(*ω*) being the magnitude of the blade point displacement; *ω* being angular frequency of cyclic load. 

#### 5.2.2. Modal Properties of a Defect-Free Blade (Noncracked)

In the case of a defect-free blade (health) resonance characteristics of particular modes were gained, ones that could be well described with a model of a single-degree-of-freedom linear system (SDOF)—of mass *m* suspended on a spring with spring rate *K* and viscous damping *C* [17–19, 23]. For sine test SDOF model describes the following relation:

(18) $$\begin{array}{c} m\frac{d^{2}y\left(t\right)}{dt^{2}}+C\frac{dy\left(t\right)}{dt}+Ky\left(t\right)=F\left(t\right),\\ F\left(t\right)=A\left(\omega \right)\sin\left(\omega t\right),\\ y\left(t\right)=B\left(\omega \right)\sin\left(\omega t+\varphi \left(\omega \right)\right). \end{array}$$

**Figure 27:**
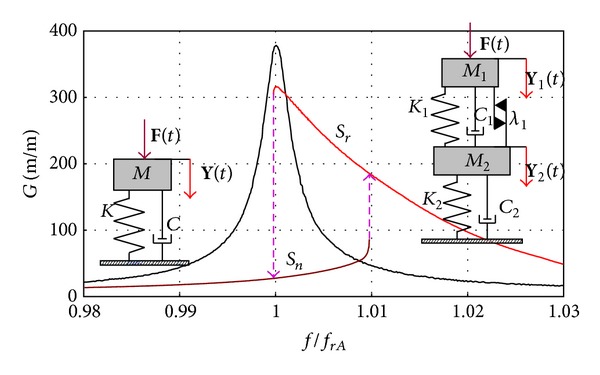
The effect of a crack on the 1st mode characteristics shape (steel blade, *a* = 19.62 m/s^2^, *f*_*rA*_: frequency of amplitude resonance) [23].

Characteristics of subsequent modes remain continuous under resonance conditions and exhibit good symmetry around the resonance frequency (within the bandwidth of 3 dB) (Figure 27) (left side). The blade displacement at the measuring point can be described as follows

(i) vibration amplitude
  (19) $$\begin{array}{c} b\left(\omega \right)=\frac{y_{\text{st}}}{\sqrt{{\left[1-{\left(\omega /\omega_{o}\right)}^{2}\right]}^{2}+{\left(\left(\delta /\pi \right)\left(\omega /\omega_{o}\right)\right)}^{2}}}, \end{array}$$
(ii) vibration phase angle
  (20) $$\begin{array}{c} \varphi \left(\omega \right)=\arctan\left(\frac{\left(\delta /\pi \right)\left(\omega /\omega_{o}\right)}{1-{\left(\omega /\omega_{o}\right)}^{2}}\right), \end{array}$$

where *ω*_0_ is a free vibration frequency, *δ* is a logarithmic damping decrement.

#### 5.2.3. Diagnostic Symptoms of a Cracked Blade

When analyzing resonance curve shape we can observe how different it is for cracked blade. The blade has all nonlinear properties [17, 21, 23] which describe a nonlinear 2DOF model (Figure 27) (right side).

**Figure 28:**
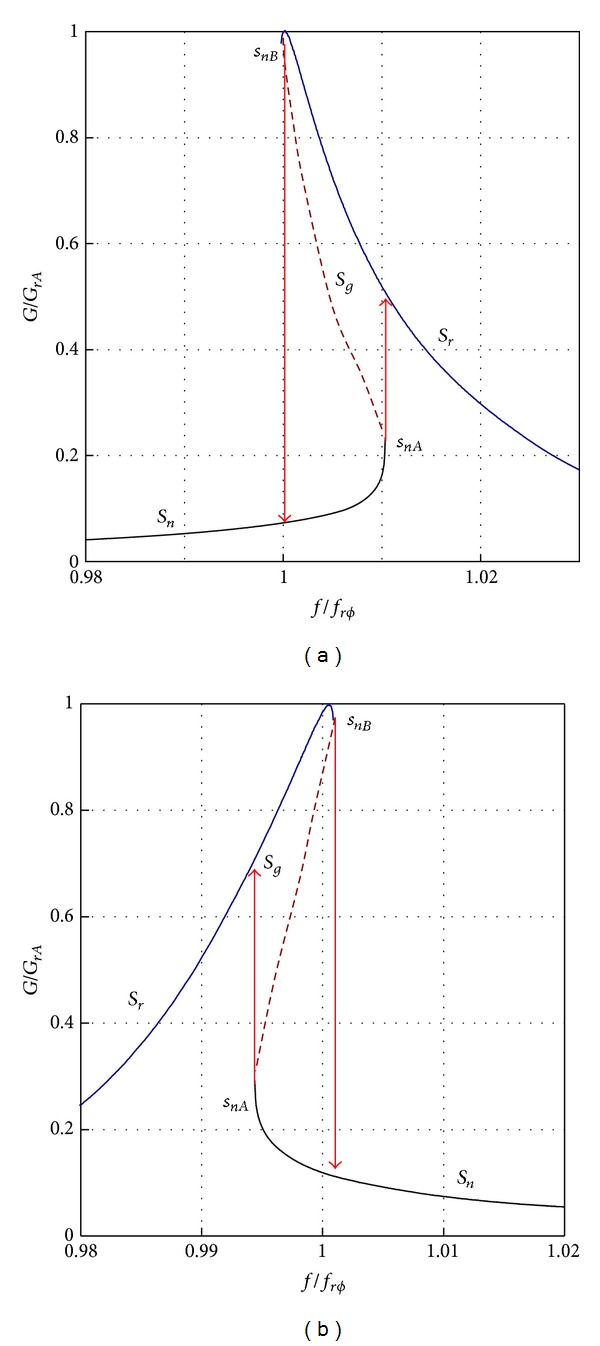
Shape of resonance characteristic for cracked blade with: (a) material weakening on the crack tip; (b) material hardening on the crack tip [3, 23].

Close to the resonance frequency it is possible to observe two branches of characteristics: resonance attractor, *S*_*r*_ (red line) and nonresonance attractor, *S*_*n*_ (blue line) and jumps between them. The shape of a crackedblade's resonant curve is affected by the blade's material and conditions existing on the edge of the crack gap (weakening or hardening, friction). The characteristic curve is sloped to the left (towards lower frequencies) for the crack with material weakening. On the other hand, for the gap with material hardening, the curve is sloped to the right (towards higher frequencies) (Figure 28). The knowledge of resonant curve inclination is essential for correct interpretation of measurements, including correct identification of the resonant and nonresonant branches. During one-sided test we observe “asymmetry” resonance curve with seeming quality factor being decreased. Resonance frequency and characteristics are functions of a blade amplitude. They were not asymmetry symptom for the following.

- Small loads that do not develop an open crack: asymmetry is growing with a load increase.
- A notch on a blade, which was used as a simplified crack model (no friction at a notch hole): no friction in notch modeled blade is a source of other differences in modal properties, Table 4, and fatigue (JCF phenomena).

The obtained characteristics of the cracked blade cannot be described with an SDOF linear model. The blade crack forms a two-degrees-of-freedom (2DOF) nonlinear system for any form of blade vibration. The equivalent linear equation that satisfies the nonlinear equation with accuracy *ε* takes the following form:

(21) $$\begin{array}{c} \frac{d^{2}y}{dt^{2}}+2h_{\varepsilon }\left(b\right)\frac{dy}{dt}+\alpha_{\varepsilon }^{2}\left(b\right)y=\varepsilon p\cos\left(\omega t\right), \end{array}$$

where *ε* is small parameter, *p* is amplitude of the exciting force, *b* the steady-state vibration amplitude, *α*_*ε*_(*b*) the equivalent natural (free-vibration) frequency, and *h*_*ε*_(*b*) the equivalent elementary damping coefficient.

The measured and analyzed parameters of the blade are described with the following relationships:

(i) vibration amplitude
  (22) $$\begin{array}{c} b\left(\omega \right)=\frac{\varepsilon p}{\sqrt{{\left(\alpha_{\varepsilon }^{2}\left(b\right)-\omega^{2}\right)}^{2}+4h_{\varepsilon }^{2}\left(b\right)\omega^{2}}}, \end{array}$$
(ii) resonance frequency
  (23) ω=(αε2(b)−2hε2(b))±d,d=4hε2(b)(hε2(b)−αε2(b))+(εpb)2,
(iii) vibration phase angle
  (24) $$\begin{array}{c} \varphi \left(\omega \right)=\arctan\left[\frac{-2h_{\varepsilon }\left(b\right)\omega }{\alpha_{\varepsilon }^{2}\left(b\right)-\omega^{2}}\right]. \end{array}$$

#### 5.2.4. Early Fatigue Identification

**Figure 29:**
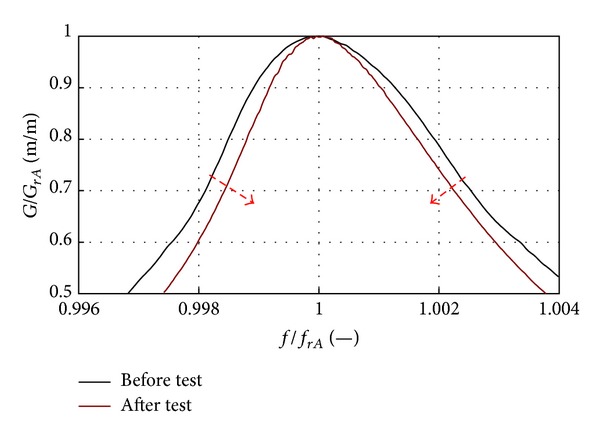
Changes in modal parameters during material strengthening phase [3, 23].

The LCF and HCF data analysis showed that blade modal properties could be used to observe the material strengthening phase [23]. Increase in the 1st mode resonance frequency of approximately 0.4% and reducing material damping are symptoms of the initial resonance system quality factor growth (correlation with structural and magnetic anisotropy) (Figure 29). This phase can be described with linear SDOF model. 

**Figure 30:**
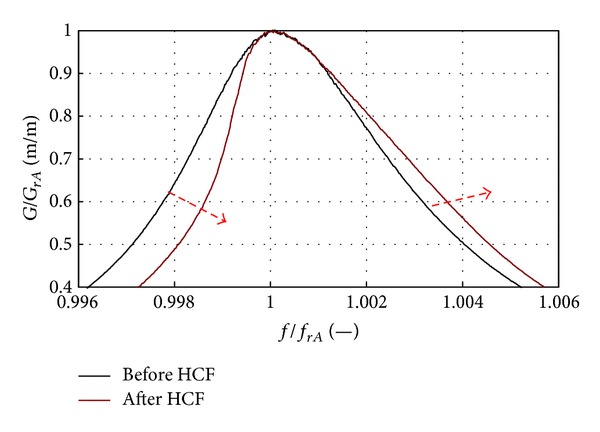
Changes in modal parameters during material weakening phase (*δf*_*rA*_ = −0.5 Hz) [3, 23].

The growing asymmetry of the resonance curve was observed only in the final fatigue phase (Figure 30); it preceded the 1st mode frequency decrease.

#### 5.2.5. JCF Phenomena

Influence of the cracked blade's resonant curve discontinuity on the propagation rate was tested for blades made from titanium alloy. It was found that in the case of constant frequency input (HCF tests without fine tuning to current resonant frequency), characteristic curve sloping to right and resonant curve discontinuity helps stopping the crack propagation.

The speed rate of its development was conditioned by the load history of a blade. The asymmetry is a symptom of the material weakening phase [3, 23]. The speed rate of the resonance curve asymmetry development, from the very first symptom of an open crack, is determined by the blade loading history.

**Figure 31:**
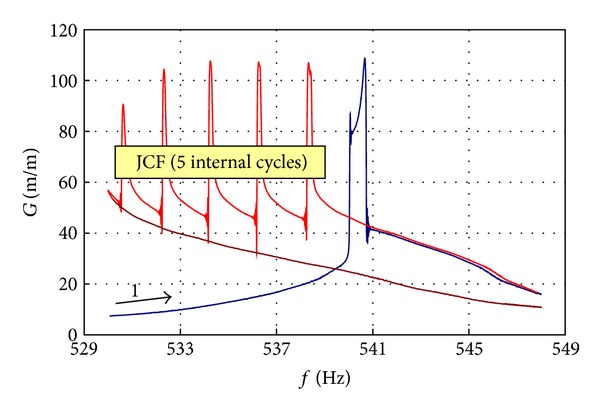
The JCF phenomenon for a cracking blade with Ti5.8Al-3.7Mo (sine sweep 4 Hz/min with constant external load) [3, 23].

Discontinuity of the resonant curve (blade pulse input discharge and load even for constant external load) is a source of very fast crack propagation during frequency transient phase—the phenomenon is called Jump Cycle Fatigue (JCF) (Figure 31). 

The JCF is a reason for the serial material tearing during the decrease in excitations frequency, observed in the unstable phase of cracking. Those observations are fundamental for the prognosis of crack propagation velocity and determination safe prognosis horizon for blade operation and fatigue reverse engineering—correct interpretation of fracture structure (answer on the question “How many load cycles took place during crack propagation?”). Arrest lines of fatigue strap map only a number of cycles for internal loads. Their values could be bigger several times than a number of cycles for external loads, which result from a flight mission profile.

### 5.3. The Tip Timing Method

The object under scrutiny has been the 1st stage compressor blade (28 blades made out of the 18H2N4WA steel, each 100 mm long, chord 37 mm, twisted by the angle of 38°). Frequencies of three subsequent modes of blade vibration were as follows (average values): 350 Hz and 1380 Hz (bending vibration), 1890 Hz (torsional vibration).

#### 5.3.1. Synchronous Resonance

**Figure 32:**
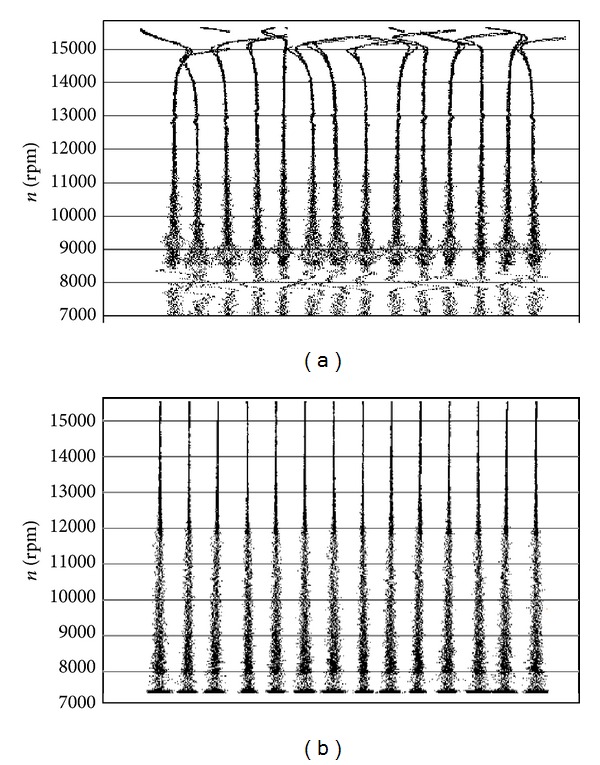
How foreign-matter depositions may affect the level of stress in the SO-3's 1st stage compressor blades [55]: (a) effect of foreign matter in compressor inlet; (b) model amplitude-phase spectra of the 1st compressor blade.

During examination with a strain gauge no evident symptoms of interrelationships between the disk and blade vibrations were observed—compressor stages are of compact design. However, it was observed that, within the take-off range of the SO-3 engine operation (*n* = 15600 rpm), synchronisation of blade vibration with forces from the 2nd harmonic of the rotational speed (*f*_1mode_ = 520 Hz) may occur (Figure 32(a)). Such phenomena observed, for example, after some foreign object (bird, ice) has been deposited on the stator blade-ring, induce blade vibration up to some dangerous level where the material yield point is reached and exceeded, and quick initiation and propagation of the LCF and HCF cracks occur. Under such conditions of blade operation, time of safe operation of any turbojet engine may be much shorter than one flight/mission of an aircraft.

#### 5.3.2. Asynchronous Resonance

**Figure 33:**
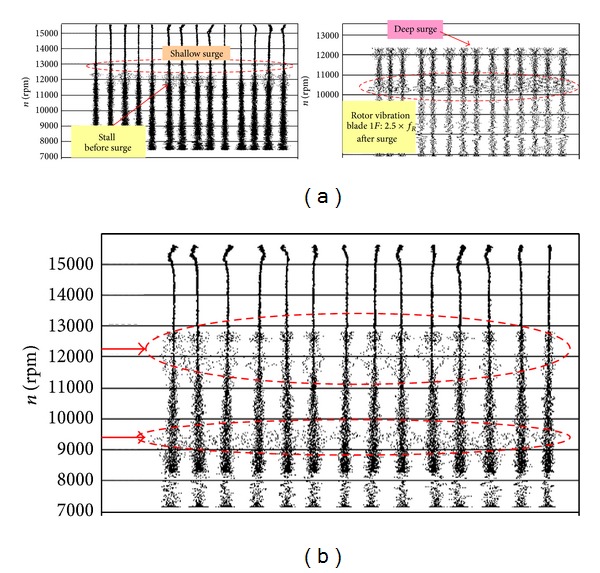
The asynchronous resonance of the compressor blades [55, 61, 63]: (a) before, during, and after the surge; (b) during identification of the surge limit (*p*_3_ signal disconnected from FCU).

The work of an engine in the compressor limits (during acceleration and deceleration of the rotation speed) exaggerated shading in the intake whether exaggerated mistakes of the rotor alignment are creating condition for the asynchronous resonance of compressor blades and HCF problems (Figure 33). They are not only endangered blades of the compressor but also other sub-assemblies of the engine, for example, bearing, gears, and shafts. The endurance risk of subassemblies mentioned above can be reduced through the engine user. For that purpose an aperiodic component TOA(*k*) is being used (phase portrait of the rotation speed) [61–63]. 

#### 5.3.3. The Blade Cracking

After an analysis of destructive testing results (controlled propagation of blade cracking under normal conditions of operating the SO-3 engine) it was found that [55]

**Figure 34:**
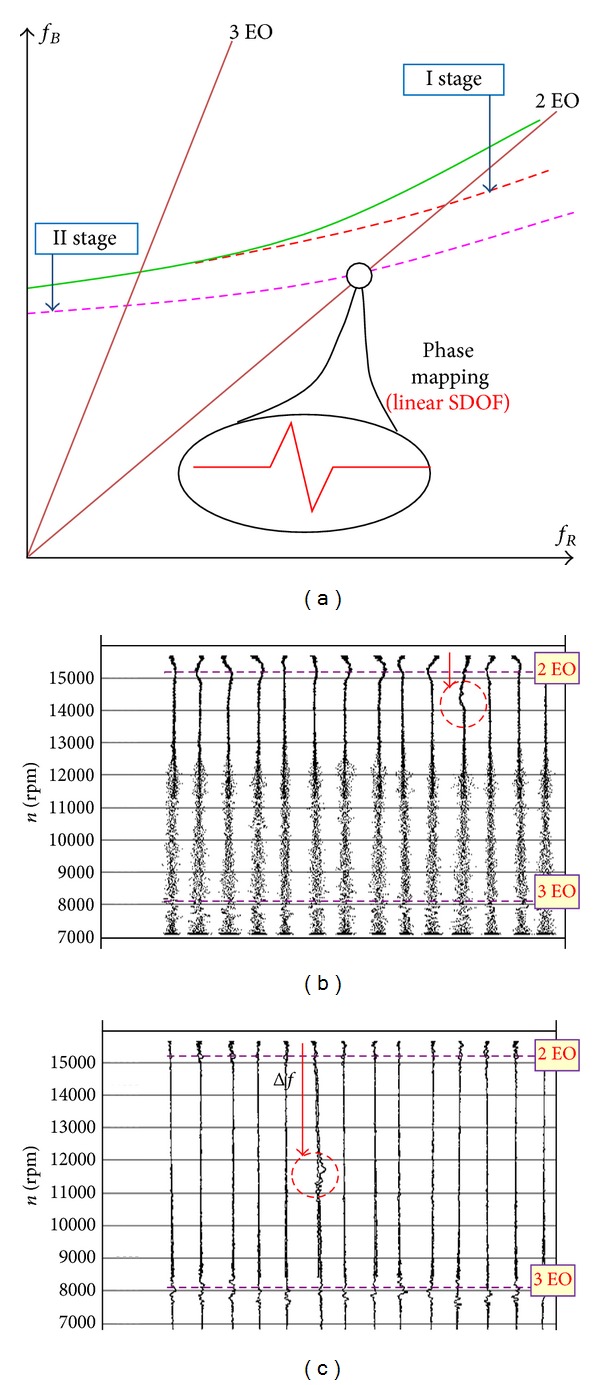
The effect of blade cracking [55]: (a) blade frequency plotted in the Campbell diagram; (b) the first stage of blade cracking, changes only *B*; (c) final stage of blade cracking, 5 minutes before break (signal after low-pass filtering).

(i) during the blade cracking initiation (no open crack visible on the blade surface) only change in the *B* factor of dynamic increment of blade vibration frequency is seen (Figure 34(a)); frequency of the blade's free vibration *f*_*B*_(0) is constant
  (25) $$\begin{array}{c} f_{B}\left(n\right)≅\sqrt{{f_{B}\left(n=0\right)}^{2}+Bn^{2}}, \end{array}$$
(ii) the occurrence of a blade crack decreases in the range of excitations from the rotational-speed II harmonic by 1000 rpm (Δ*f* = 16.6 Hz) (Figure 34(b)). At the moment, frequency (the 1st mode) of the blade's free vibration changed by less than 3 Hz;
(iii) when the crack reaches about 30% of the blade profile, evident reduction in frequency of free vibration and decrease in the range of excitations from the rotational-speed III harmonic (*n*≅8000 rpm) were observed;
(iv) just before the blade break-off (about 65% of profile for the crack from the leading edge, 95% of profile for the crack from the back of the blade), an evident effect of stiffening due to centrifugal forces was observed (Figure 34(c)). Changes in the dynamic scale inflicted by the broken blade are comparable with those in other dynamic scales (the influence of the engine's rotational speed).

It has been proven that the TTM gives credible prognosis for 50 engine work hours—“over 9 · 10^7^ HCF and 100 LCF cycles, 1/8 TBO [3, 23, 62].” It has been also proven that TTM symptoms of the cracking are closely related to

- *the strengthening phase*: the quality factor of the resonance system increases together with the friction mode frequency;
- *the weakening phase*: growth in the resonance curve asymmetry and growth in nonlinearity.

## 6. Conclusion

1. Described methods are mutually supplementing, which results in the synergy effect. The verified knowledge enables better modeling of continuum damage mechanisms and improving research method.
2. New opportunities of the metal magnetic memory method, including diagnosing and identification of hidden risk of material fatigue (before the opened crack, measurements through the casing, and SHM application), have been proven.
3. The high effectiveness of the experimental modal analysis method has been demonstrated on the basis of tests of more than 3000 compressor blades. A possibility of the automatic detection of the crack, the weakness (fatigue softening) and strengthening (strain hardening) of the blade, has been presented. The shape of resonance curve is diagnostic symptom.
4. Active control of blade fatigue by the aeroengine user is possible. During 20 years of using the tip timing method in the Armed Forces of Poland, the following things have occurred.
  -  The statistical mean time between fatigue break-offs of blades has been increased (eleven times for calendar-based data and seven times on the hourly basis). Since 1991 the fatigue crack of any compressor blade in the SO-3 engines has not been registered in spite of the existing fault in design.
  -  The surge as a result of maladjustment of the fuel system and latent defects of subsystems has been eliminated (mainly fatigue problems results from maintenance).
  -  Five SO-3 engines have been taken out of service due to excessive errors in shapes of the blades.
5. Nonlinear properties of a crack blade are fundamental for the prognosis of the crack propagation rate and for the determining safe prognosis horizon. The modal symptoms of material damage are correlated with magnetic symptoms. Asymmetry of resonant curve has not been found on the blade with notched-a simple crack simulation model, often found in the literature.

## Figures and Tables

**Table 1:** Endurance threats of real blades permanence (“live”) according to the theory of five elements [3].

Flow dynamics path	Kinematic loads path
Flow clocking Stall Surge Flutter Combustion instability Foreign object in inlet Flight on big angles of the attack	Centrifugal force Compressor nonaxial Rotor unbalance Compressor speed fluctuation Structure resonances Flight with big “g”

**Table 2:** The fatigue of metal compressor blades at numbers of load cycles in the range 10^0^–10^12^ [69].

Number of cycles	Description
10^0^–10^3^	Low cycle quasi-static or fatigue fracture (LCF problem) at availability of large “microplastic” deformation in some zones of failure. The crack nucleation below the surface.
10^3^–10^5^	Low cycle fatigue fracture (LCF problem) at availability rather small “macroplastic” deformation in a zone of failure (when *σ*_*e*_ ≤ *σ*_*a*_ ≤ *σ*_*y*_, *σ*_e_—a limit of elasticity). The crack nucleation from the surface.
10^5^–10^8^	Many classical cycles fatigue fracture (HCF problem) at availability of “microplastic” deformations in micro and macrovolumes near a zone of fatigue (when *σ*_*a*_≤*σ*_*e*_). The crack nucleation from the surface.
10^9^–10^12^	Fatigue fracture on super high bases (VHCF problem) at availability of “microplastic” deformation in microvolumes near a zone of failure (when *σ*_*a*_ ≪ *σ*_*e*_). The crack nucleation on the soft inclusion below the surface.

**Table 3:** Potential field of tip timing method use.

Object	Transmitter of putting the angle turnover
Wind turbine	Blade
Helicopter	Main rotor blade Tail rotor blade
Propeller aircraft	Propeller blade
Power turbine	Turbine blade
Jet engine	Compressor blade Turbine blade
Turboprop	Compressor blade Turbine blade
ABS systems, gearbox	Gear wheel
engine control unit	Gear wheel
bearings	Rolling element

**Table 4:** Blade with 11 mm length damage (starting from TE) placed 20 mm from lock [23, 25].

Blade	Frequency change (Hz)		
	1st mode	2nd mode	3rd mode
Cracked	−12	+7	−27
Notched (no friction)	−13	−5	−80
Difference (%)	**−0.28**	**−0.86**	**−2.73**

## References

[1] More intelligent gas turbine engines. http://www.cso.nato.int/pubs/rdp.asp?RDP=RTO-TR-AVT-128.

[2] Active control of engine dynamics. http://www.cso.nato.int/Pubs/rdp.asp?RDP=RTO-EN-020.

[3] Witoś M (2011). Increasing the durability of turbine engines through active diagnostics and control. *Research Works of AFIT*.

[4] Vlasov VT, Dubov AA (2004). *Physical Bases of the Metal Magnetic Memory Method*.

[8] Dubov A, Kolokolnikov S (2013). The metal magnetic memory method application for online monitoring of damage development in the steel pipes and welded joints specimens. *Welding in the World*.

[9] Yan K, Wang ZD, Deng B, Shen K (2012). Experimental research on metal magnetic memory method. *Experimental Mechanics*.

[10] Roskosz M Metal magnetic memory testing of welding joints of ferritic and austenitic steels.

[11] Ding X, Li J, Li F, Pang X Magnetic memory inspection of high pressure manifoolds.

[12] Liu Q, Lin J, Chen M A study of inspecting the stress on downhole metal casing in oilfields with magnetic memory method.

[13] Hai Yan X, Qiang XM, Zhijun Y, Lihong Z Stress state Analysis of failure blade with MMM method. http://www.paper.edu.cn/.

[14] Iwaniec M, Witoś M, Roskosz M, Gontarz S Diagnosis of bearer structures of high voltage lines using magneto-mechanical effects.

[15] Witoś M (2012). The reference signal of geomagnetic field for MMM expert systems. *Key Engineering Materials*.

[16] Witoś M, Zieja M (2011). High sensitive methods for fatigue detection. *Diagnostyka*.

[17] Maia NMM, Silva JMM (1997). *Theoretical and Experimental Modal Analysis*.

[18] Ewins DJ (2000). *Modal Testing: Theory, Practice and Application*.

[19] Heylen W, Lammens S, Sas P (1997). *Modal Analysis Theory and Testing*.

[20] Schwarz BJ, Richardson MH (1999). *Experimental Modal Analysis*.

[21] Ostrovsky LA, Johnson PA (2001). Dynamic nonlinear elasticity in geomaterials. *Revista Del Nuovo Cimento*.

[22] Witoś M, Stefaniuk M (2010). Compressor blade fatigue diagnostic and modeling with the use of modal analysis. *Fatigue of Aircraft Structure*.

[23] Witoś M (2008). On the modal analysis of a cracking compressor blade. *Research Works of AFIT*.

[24] Report on aircraft PP-VNN. http://aviation-safety.net/database/.

[25] Brouckaert JF (2007). *Tip Timing and Tip Clearance Problems in Turbomachinary*.

[26] http://www.agilismeasurementsystems.com/.

[27] Ayes BW, Arnold S, Vining C, Howard R Application of generation 4 non-contact stress measurement system on HCF demonstrator engines.

[28] Duan F-J, Fang Z-Q, Sun Y-Y, Ye S-H (2005). Real-time vibration measurement based on tip-timing for rotating blades. *Opto-Electronic Engineering*.

[29] von Flotow A, Drumm MJ (2002). *Engine Sensing Technology Hardware and Software to Monitor Engine Rotor Dynamics Using Blade Time-of-Arrival and Tip Clearance*.

[30] Przysowa R, Spychala J (2008). Health monitoring of turbomachinery based on blade tip-timing and tip-clearence. *RTO-MP-AVT-157*.

[31] Washburn R Amplitude and phase variations associated with low order resonance responses subjected to time varying excitation sources.

[32] Witoś M, Uhl T, Ostachowicz W, Holnicki-Szulc J Turbine engine health/maintenance status monitoring with use of tip timing method.

[33] Zielinski M, Ziller G (2005). Noncontact crack detection on compressor rotor blades to prevent further damage after HC-failure. *RTO MP-AVT-121*.

[34] Campbell W (1924). Elastic-fluid turbine rotor and method of avoiding tangential bucket vibration therein. *Patent US*.

[35] http://www.evi-gti.com/.

[36] http://www.piwg.org/.

[37] Hardigg GH, Swarthmore PA (1951). Apparatus for measuring rotor blade vibration. *Patent US*.

[38] Shapiro H (1962). Vibration detector and measuring instrument. *Patent US*.

[39] Zablotsky IE, Korostelev JA, Lebedev AY, Sviblov LB, Tolchinsky EM (1969). Vibrator indicator for turboengine rotor blading. *Patent US*.

[40] Smejkal J, Jindra M, Brezina Z (1971). Apparatus for switching pulses in measuring the vibration of rotating parts during operation of a machine. *Patent US*.

[41] Naegeli J, Maurer A (1979). Method and apparatus for monitoring the state of oscillation of the blades of a rotor. *Patent US*.

[42] Ellis VEH (1986). Vibration monitoring in rotary machines. *Patent US*.

[43] Marron GI, Rethage WB (1989). Blade pitch measurement apparatus and method. *Patent US*.

[44] McKendree FS, Rozelle PF (1989). Nonsynchronous turbine blade vibration monitoring system. *Patent US*.

[45] Kending RP, Lucheta RA, McKendree FS (1990). Turbine blade fatigue monitor. *Patent US*.

[46] Kudelski R, Szczepanik R (1991). System for signalling the fact of exceeding admissible amplitude of vibrations by vanes of a fluid-flow machine. *Patent*.

[47] Twerdochlib M, Rozelle PF, Sarasas S (1992). Apparatus and method for removing common mode vibration data from digital turbine blade vibration data. *Patent US*.

[48] Witoś M, Gawin A, Szczepankowski A (1996). Sposob diagnozowania technicznego wirujacych lopatek maszyny wirnikowej oraz uklad do diagnozowania technicznego wirujacych lopatek maszyny wirnikowej. *Patent PL*.

[49] Kendig RP, Lucheta RA, McKendree FS (1996). Turbine blade fatigue monitor. *Patent*.

[50] Yukio M, Masanori E (2000). Vibration measuring apparatus for rotor blade. *Patent*.

[51] Mednikov VA, Shchegolev VV (2003). Method for determining amplitude of oscillations of turbomachine blade. *Patent*.

[52] Hideyasu I, Kenichi N, Shinya M (2005). Moving blade failure diagnosing method of gas turbine and device therefor. *Patent*.

[53] Shchegolev VV (2005). Method for metering vibration amplitudes of turbomachine blades. *Patent*.

[54] Zielinski M, Ziller G (2010). Method and device for detecting cracks in compressor blades. *Patent*.

[55] Witoś M (1994). *Diagnosing of technical condition of turbine engine compressor blades using non-contact vibration measuring method [Ph.D. thesis]*.

[56] Shaniavski AA (2007). *Modeling of Fatigue Cracking of Metals. Synergetics For Aviation*.

[57] Shaniavski AA (2003). *Tolerance Fatigue of Aircraft Components. Synergetics in Engineering Applications*.

[58] Murakami Y, Nomoto T, Ueda T (1999). Factors influencing the mechanism of superlong fatigue failure in steels. *Fatigue and Fracture of Engineering Materials and Structures*.

[59] Murakami Y, Takada M, Toriyama T (1998). Super-long life tension-compression fatigue properties of quenched and tempered 0.46% carbon steel. *International Journal of Fatigue*.

[60] Sakai T (2009). Review and prospects for current studies on very high cycle fatigue of metal materials for machine structural use. *Journal of Solid Mechanics and Materials Engineering*.

[61] Szczepankowski A (1999). *Diagnosing of Technical Condition of Turbine Engine Using Rotational Speed Phase-Mapping Method [Ph.D. thesis]*.

[62] Szczepanik R, Witoś M Aeroengine condition monitoring system based on non-interference discrete-phase compressor blade vibration measuring method. http://ftp.rta.nato.int/public//PubFullText/RTO/MP/RTO-MP-051///MP-051-PSP-13.pdf.

[63] Kowalski M (2012). Phase mapping in the diagnosing of a turbojet engine. *Journal of Theoretical and Applied Mechanics*.

[64] Kocanda S (1985). *Zmeczeniowe Pekanie Metali*.

[65] Almojil MA (2010). *Deformation and recrystal-lisation in low carbon steels [Ph.D. thesis]*.

[66] Novikov V (1996). *Grain Growth and Control of Microstructure and Texture in Polycrystalline Materials*.

[67] Cottrell AH (1964). *The Mechanical Properties of Matter*.

[68] Nabarro FRN (1947). Dislocations in a simple cubic lattice. *Proceedings of the Physical Society*.

[69] Makhutov NA, Gadenin MM Nonlinear deformation and fracture mechanics for engineering approaches in design of structure. http://www.eolss.net/.

[70] Cui W (2002). A state-of-the-art review on fatigue life prediction methods for metal structures. *Journal of Marine Science and Technology*.

[71] Socha G (2003). Experimental investigations of fatigue cracks nucleation, growth and coalescence in structural steel. *International Journal of Fatigue*.

[72] Pickering FB (1978). *Physical Metallurgy and the Design of the Steels*.

[73] Thompson SM (1991). *The magnetic properties of plastically deformed steels [Durham theses]*.

[74] Lee T-K, Morris JW, Lee S, Clarke J Detection of fatigue damage prior crack initiation with scanning SQUID microscopy.

[75] Newnham R (2005). *Properties of Materials. Anisotropy, Symmetry, Structure*.

[76] Vandenbossche L (2009). *Magnetic Hysteretic Characterization of Ferromagnetic Materials with Objectives towards Non-Destructive Evaluation of Material Degradation [Ph.D. thesis]*.

[77] Ewing JA (1900). *Magnetic Induction in Iron and Other Metals*.

[78] Yaegashi K (2007). Dependence of magnetic susceptibility on dislocation density in tensile deformed iron and Mn-steel. *ISIJ International*.

[79] Liang W, Fang D, Shen Y, Soh AK (2002). Nonlinear magnetoelastic coupling effects in a soft ferromagnetic material with a crack. *International Journal of Solids and Structures*.

[80] Burrows CW Correlation of the magnetic and mechanical properties of steel. http://www.archive.org/.

[81] Blanter MS, Golovin IS, Neuhäuser H, Sinning HR (2007). *Internal Friction in Metallic Materials. A Handbook*.

[82] Fischer MF (1928). Note on the effect of repeated stresses on the magnetic properties of steel. *Bureau of Standards Journal of Research*.

[83] Robertson IM (1991). Magneto-elastic behaviour of steels for naval applications. *MRL Technical Report*.

[84] Yamasaki T, Yamamoto S, Hirao M (1996). Effect of applied stresses on magnetostriction of low carbon steel. *NDT and E International*.

[85] Atherton DL, Jiles DC (1986). Effects of stress on magnetization. *NDT International*.

[86] Birss RR, Faunce CA (1971). Stress-induced magnetization in small magnetic fields. *Journal de Physique*.

[87] Lee EW (1955). Magnetostriction and magneto-mechanical effects. *Reports on Progress in Physics*.

[88] Smith CM, Sherman GW (1914). A study of the magnetic qualities of stressed iron and steel. *Physical Review*.

[89] http://www.magnaflux.com/.

[90] http://www.magneticsensors.com/.

[91] http://www.energodiagnostika.ru/.

[92] http://polytec.com/.

[93] http://www.bksv.com/.

[94] http://www.mtiinstruments.com/.

[95] http://www.vibrationresearch.com/.

[96] http://www.acam-usa.com/.

[97] http://highlandtechnology.com/.

